# Genomic Networks of Hybrid Sterility

**DOI:** 10.1371/journal.pgen.1004162

**Published:** 2014-02-20

**Authors:** Leslie M. Turner, Michael A. White, Diethard Tautz, Bret A. Payseur

**Affiliations:** 1 Max Planck Institute for Evolutionary Biology, Evolutionary Genetics, Ploen, Germany; 2 University of Wisconsin, Laboratory of Genetics, Madison, Wisconsin, United States of America; Duke University, United States of America

## Abstract

Hybrid dysfunction, a common feature of reproductive barriers between species, is often caused by negative epistasis between loci (“Dobzhansky-Muller incompatibilities”). The nature and complexity of hybrid incompatibilities remain poorly understood because identifying interacting loci that affect complex phenotypes is difficult. With subspecies in the early stages of speciation, an array of genetic tools, and detailed knowledge of reproductive biology, house mice (*Mus musculus*) provide a model system for dissecting hybrid incompatibilities. Male hybrids between *M. musculus* subspecies often show reduced fertility. Previous studies identified loci and several X chromosome-autosome interactions that contribute to sterility. To characterize the genetic basis of hybrid sterility in detail, we used a systems genetics approach, integrating mapping of gene expression traits with sterility phenotypes and QTL. We measured genome-wide testis expression in 305 male F_2_s from a cross between wild-derived inbred strains of *M. musculus musculus* and *M. m. domesticus*. We identified several thousand *cis*- and *trans*-acting QTL contributing to expression variation (eQTL). Many *trans* eQTL cluster into eleven ‘hotspots,’ seven of which co-localize with QTL for sterility phenotypes identified in the cross. The number and clustering of *trans* eQTL—but not *cis* eQTL—were substantially lower when mapping was restricted to a ‘fertile’ subset of mice, providing evidence that *trans* eQTL hotspots are related to sterility. Functional annotation of transcripts with eQTL provides insights into the biological processes disrupted by sterility loci and guides prioritization of candidate genes. Using a conditional mapping approach, we identified eQTL dependent on interactions between loci, revealing a complex system of epistasis. Our results illuminate established patterns, including the role of the X chromosome in hybrid sterility. The integrated mapping approach we employed is applicable in a broad range of organisms and we advocate for widespread adoption of a network-centered approach in speciation genetics.

## Author Summary

New species are created when barriers to reproduction form between groups of organisms that formerly interbred freely. Reduced fertility or viability of hybrid offspring is a common form of reproductive isolation. Hybrid defects are caused by negative interactions between genes that have undergone evolutionary change within each subgroup. Identifying genetic interactions causing disease or trait variation is very difficult, consequently there are few known hybrid incompatibility genes and even fewer cases where both interacting genes are known. Here, we combined mapping of gene expression levels in testis with previous results mapping male sterility traits in hybrid house mice. This new approach to finding genetic causes of reproductive barriers enabled us to identify a large number of hybrid incompatibilities, involving genomic regions with known roles in hybrid sterility and previously unknown regions. Understanding the number and type of genetic interactions is important for developing accurate models used to reconstruct speciation events. The genetics of hybrid sterility in mice may also contribute to understanding basic processes involved in male reproduction and causes of human infertility.

## Introduction

To understand patterns of biodiversity, it is essential to characterize the processes by which new species arise and are maintained in nature, including ecological specialization, population differentiation and reproductive isolation. Genetic dissection of reproductive isolation has proven to be an especially powerful strategy for revealing mechanisms of speciation. Many genomic regions and even specific genes that contribute to hybrid defects have been identified by genetic mapping in recombinant populations [1]–[7]. Divergence in gene regulation is expected to contribute to reproductive isolation between nascent species, and studies with F_1_ hybrids support this prediction [8]–[13]. Importantly, these two approaches – genetic mapping and measurement of genome-wide expression patterns in hybrids – have yet to be combined directly in the context of speciation.

Hybrid sterility and hybrid inviability frequently result from negative epistasis between mutations at interacting genes [14]–[16]. This “Dobzhansky-Muller model” predicts that disruptions in gene networks should be common in hybrids. By integrating organismal phenotypes and genotypes with gene expression patterns, this prediction can be tested. Despite the identification of hybrid incompatibility genes in several species and the prevalence of the Dobzhansky-Muller model, the nature and complexity of hybrid incompatibility networks remains poorly understood. Do hybrid incompatibilities generally involve two loci or are higher order interactions common? Are incompatibilities independent or do they share some common loci? Is the genetic architecture of hybrid defects similar among taxa? Known incompatibility genes have provided the first hints about these questions, particularly in *Drosophila* [6], yet too few genes and taxa are represented to determine whether there are generalities underlying the speciation process. A network perspective should provide insights into the genetics of reproductive isolation that are difficult to obtain using a gene-by-gene approach.

The house mouse (*Mus musculus*) is an excellent model for investigating speciation from a network perspective. Genomic resources are abundant for the house mouse, and reproductive processes are well characterized because the mouse is the premier model for fertility research in humans [17]. House mouse subspecies are in the early stages of speciation, showing significant but incomplete reproductive isolation. Evidence for hybrid male sterility in laboratory crosses [5], [18]–[22] and in natural zones of hybridization [23], [24] suggests it is a primary isolating barrier between these nascent species.

Studies of sterility in F_1_ hybrids between *Mus musculus domesticus* and *Mus musculus musculus* (subsequently referred to as *domesticus* and *musculus*) revealed an important role for the X chromosome and identified several contributing autosomal loci [4], [5], [25], [26]. One of these loci is *Prdm9*, a histone methyltransferase [27]. Hybrids with some alleles of *Prdm9* from *domesticus* show pachytene arrest of meiosis. The effects of sterile *Prdm9* alleles appear to be due to mutations in the protein-coding sequence and there is evidence for downstream regulatory effects, but the incompatibility network involving *Prdm9* has not been revealed.

Genetic mapping of sterility phenotypes in F_2_ hybrids between *M. m. domesticus* and *M. m. musculus* recently identified an additional set of autosomal loci, which are predominantly recessive and thus contribute to sterility in second generation and subsequent hybrids. Genetic architectures of F_2_ sterility traits are complex, involving a moderate number of loci with a range of phenotypic effect sizes [1].

Genome-wide studies of gene expression in testis of F_1_ hybrids provide evidence that sterility is associated with disrupted expression [9], [10]. Like sterility phenotypes, expression patterns in hybrids depend on the origins of parental strains, and the direction of the cross. In many cases, testis expression in hybrids is intermediate between parental strains [9]–[11]. However, extensive misexpression (expression outside the range observed in parental strains) has been documented in a few crosses. Comparison of testis gene expression patterns between reciprocal F_1_ *musculus-domesticus* hybrids showed that many X-linked genes are overexpressed in sterile but not in fertile F_1_s [10]. To our knowledge, gene expression patterns in testes from F_2_ and later generation hybrids have not been described.

Here, we integrate analysis of genome-wide expression in testis from F_2_ *musculus-domesticus* hybrids with results from a previous study mapping sterility phenotypes in the same individuals [1]. We show that sterility is associated with large-scale alterations in gene expression in F_1_s and F_2_s, and we identify quantitative trait loci (QTL) that cause X chromosome-wide overexpression in hybrids. We report expression quantitative trait loci (eQTL) for a large number of transcripts. We compare the locations of eQTL with sterility QTL, and identify disrupted processes during spermatogenesis based on affected networks. Using a conditional mapping approach, we pinpoint genetic interactions affecting expression. We highlight candidate pathways, processes, and interactions for several loci, which provide insight into the mechanisms underlying their contributions to sterility.

## Results

### Gene misexpression in testes of F_1_ and F_2_ hybrids

We measured levels of misexpression in F_1_ and F_2_ hybrids to identify major alterations in gene expression pattern associated with sterility in *M. m. domesticus* (WSB/EiJ; hereafter *domesticus* ^WSB^) - *M. m. musculus* (PWD/PhJ; hereafter *musculus* ^PWD^) hybrids. Sterility is asymmetric in these crosses: F_1_ males with *musculus* ^PWD^ mothers (hereafter MxD F_1_s) are almost always completely sterile whereas F_1_s with *domesticus* ^WSB^ mothers (hereafter DxM F_1_s) are fertile [1]. MxD F_1_ males showed significant differences from both parents for all reproductive traits measured. By contrast, all traits in DxM F_1_s (except seminiferous tubule area) were within the range observed in the parental lines. Trait measurements in MxD F_1_s and DxM F_1_s provide ‘fertile’ and ‘sterile’ examples that are useful for assessing trait distributions in F_2_s.

**Figure 1:**
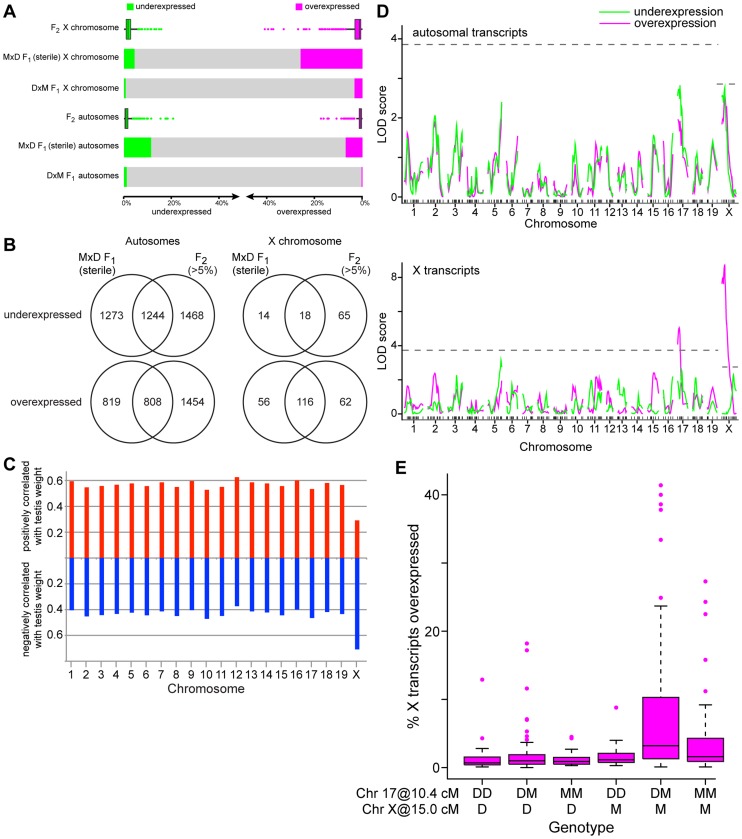
Misexpression in testes of F_1_ and F_2_ hybrids. (A) Proportions of underexpressed (green) and overexpressed (magenta) transcripts in fertile (DxM) F_1_s, sterile (MxD) F_1_s, and F_2_s. Mean values are shown for F_1_s and boxplots for F_2_s indicate median, interquartile range, and outliers >1.5× interquartile range are shown as points. (B) Proportions of misexpressed transcripts common to sterile (MxD) F_1_s and F_2_s. (C) Columns indicate proportions of transcripts on each chromosome significantly positively (red, upward) and negatively (blue, downward) correlated with right relative testis weight. (D) QTL mapping of misexpression (number of under- or over-expressed transcripts/individual) in F_2_s. Significance thresholds, determined by permutation, are indicated with dashed lines. (E) Overexpression of X-linked transcripts in F_2_s by two-locus genotype for chromosomes 17 (10.4 cM) and X (15.0 cM). Boxes indicate interquartile range, horizontal lines indicate medians, and whiskers extend to 1.5× interquartile range. Outliers are indicated with points.

Misexpression was markedly higher in testis of MxD F_1_s (18.8% transcripts; Fig. 1A) than in DxM F_1_s (1.6%). In both F_1_s, levels of misexpression were higher for X-linked transcripts than autosomal transcripts. On the X chromosome, the number of overexpressed transcripts in MxD F_1_s was much higher than the number of underexpressed transcripts (25.9% over, 4.4% under). The level of underexpression was higher on autosomes, but the difference between levels of over- and underexpression was smaller (7.1% over, 11.3% under). These results are consistent with previously reported differences in expression patterns between sterile and fertile F_1_s [10].

Misexpression in F_2_s varied from 0.9–39.0% transcripts (median 2.1%; Fig. 1A), encompassing the levels observed in fertile and sterile F_1_s. There was substantial overlap between transcripts misexpressed in MxD F_1_s and in >5% of F_2_s (Fig. 1B) yet a large proportion of transcripts were misexpressed only in F_1_s or F_2_s. The relatively continuous distribution of misexpression in F_2_s and lack of recapitulation of the full F_1_ misexpression pattern indicates multiple genetic factors contribute to misexpression. Misexpression unique to F_2_s suggests some contributing loci act recessively.

A large proportion of X-linked transcripts were negatively correlated with testis weight (lower testis weight = higher expression) – opposite of the pattern for autosomal transcripts, a majority of which was positively correlated with testis weight (Fig. 1C). This result suggests that – as in sterile F_1_s – the X may be broadly overexpressed in sterile F_2_s.

To determine whether the level of misexpression was consistent throughout spermatogenesis, we compared patterns of expression in F_1_ and F_2_ hybrids among genes identified as specific/enriched to different spermatogenic cell types in previous studies [28]. Autosomal transcripts expressed in meiotic and post-meiotic cells are underexpressed in sterile MxD F_1_s, and transcripts specific to somatic and mitotic cells are overexpressed (Table S1). This pattern is consistent with reduced spermatogenesis, as expected based on sterility phenotypes. The X chromosome is transcriptionally silenced during meiosis (meiotic sex chromosome inactivation MSCI; [29], [30]), and thus lacks transcripts associated with meiotic cells. X-linked transcripts associated with other testis cell types showed patterns consistent with autosomal transcripts; somatic and mitotic transcripts tended to be overexpressed and the few underexpressed transcripts were predominantly postmeiotic. Misexpression patterns across spermatogenic cell types in F_2_ hybrids were consistent with patterns in sterile F_1_s.

#### Misexpression QTL

Our experimental design allowed us to map QTL that contribute to misexpression in F_2_s. We identified two QTL controlling the number of overexpressed X-linked transcripts on chromosomes 17 (8 cM, 18.46 Mb, 1.5-LOD interval 0–29.85 Mb; Fig. 1D) and X (14.98 cM, 61.02 Mb, 1.5-LOD interval 0–71.69 Mb). There were no significant QTL for the number of misexpressed autosomal transcripts. However, on both chromosomes 17 and X, there were peaks below the significance threshold within the 1.5-LOD intervals of the X-overexpression QTL. Similarity between X- and autosomal patterns suggests these QTL may contribute to misexpression genome-wide.

The overexpression QTL we identified on the X chromosome is in the same region associated with overexpression of a set of X-linked transcripts in a recent study using introgression lines carrying regions of the X chromosome from *musculus* ^PWK^ (closely related to *musculus* ^PWD^) on a *domesticus* background [31]. Studies of F_1_ hybrid sterility have identified a key incompatibility between chromosomes 17 and X [4]. A comparison of X overexpression levels for mice with different two-locus genotypes at the chromosome 17 and X QTL is shown in Figure 1E. Consistent with the pattern in F_1_s, X overexpression was highest in individuals with a *musculus* ^PWD^ allele on the X that were heterozygous at the chromosome 17 QTL (F_5,290_ = 11.06, *p* = 9.2×10^−10^; t 17^het^:X*^musculus^* interaction term = 3.0, *p* = 0.0031). Interestingly, high levels of overexpression were also observed in individuals with the *musculus* ^PWD^ allele on the X and homozygous for the *musculus* ^PWD^ chromosome 17 allele. These mice had the same genotype as the *musculus* ^PWD^ parental strain at both loci, implying the existence of a *domesticus* ^WSB^ allele elsewhere in the genome involved in a multilocus interaction with the X and 17 QTL.

Interpreting gene expression patterns from whole testis is complicated because differences in measured expression levels reflect changes in the relative cell-type composition of the tissue in addition to changes in per-cell expression levels [10]. Because the number of postmeiotic cells in sterile F_2_ hybrids was reduced [1], apparent underexpression of postmeiotic genes and overexpression of mitotic genes was expected. Misexpression patterns may be caused by changes in cell composition, changes in gene regulation, or both.

### Testis expression quantitative trait loci (eQTL)

Next, we investigated the genetic basis of gene expression variation in individual transcripts. We identified 16,705–36,753 eQTL, depending on the significance criterion (Table 1). We used a permissive threshold, based on permutation of a single transcript, for downstream analyses because our goal was to identify genome-scale patterns. It is important to note that the false-positive rate among individual eQTL identified using this criterion is high, particularly for *trans* eQTL.

**Table 1:** Expression quantitative trait loci (eQTL).

Significance criterion	LOD threshold autosomes	LOD threshold X	*cis* 1 eQTL autosomes	*cis* 1 eQTL X	*cis* 1 eQTL total	*trans* 2 eQTL autosomes	*trans* 2 eQTL X	*trans* 2 eQTL total
**permutation, single transcript**	3.7	2.9	14,332	475	14,807	12,347	9,599	21,946
** 10% FDR**	4.74	2.89	13,337	475	13,812	4,930	9,633	14,563
** 5% FDR**	4.98	3.23	13,143	464	13,607	4,076	8,649	12,725
**permutation, dataset**	8.01	5.56	11,118	402	11,520	719	4,466	5,185

1 eQTL peak <5 cM from probe.

2 eQTL peak on different chromosome from probe.

**Figure 2:**
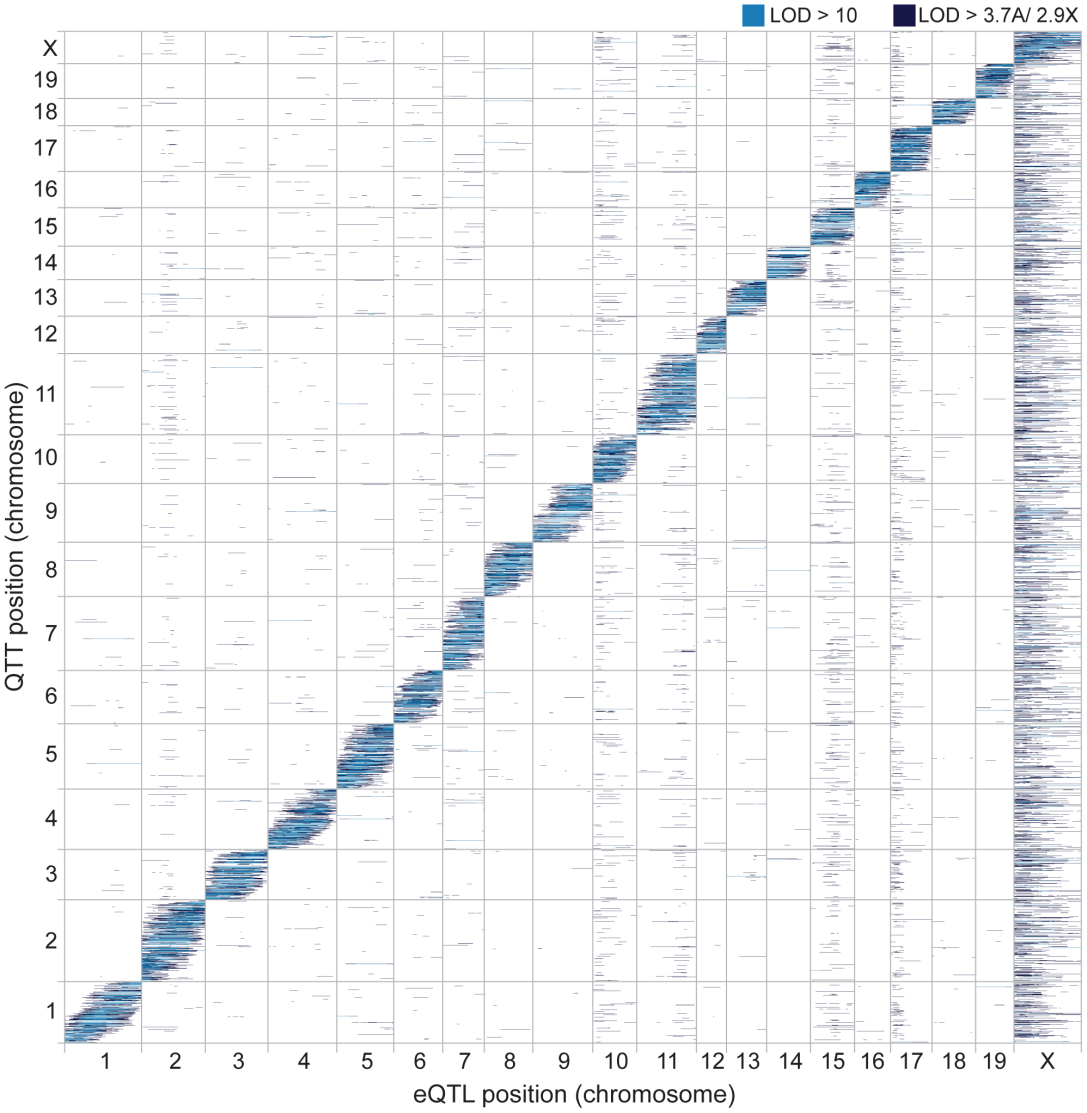
Genomic distribution of eQTL and QTT. Heatmap showing eQTL locations (marker/pseudomarker positions on x-axis) and transcript locations (y-axis). LOD scores above permutation thresholds are shown in dark blue and LOD scores >10 in light blue.

The genomic positions of the eQTL and the affected transcripts are shown in Figure 2. eQTL located near the quantitative trait transcript (QTT) comprise the prominent diagonal stripe, a pattern typical of eQTL studies [32]–[34]. These proximal eQTL are likely to be *cis* regulatory elements [33], [35]. We refer to proximal eQTL as *cis* eQTL for convenience, although it is possible that they might not act solely in *cis* (by regulating alleles only if they are on the same DNA strand). We classified eQTL with peaks within 5 cM of the transcript (probe) position as *cis* eQTL and eQTL located on a different chromosome from the transcript as *trans*. We ignored eQTL>5 cM on the same chromosome, because this class might include long-distance *cis* eQTL in addition to *trans* eQTL. We identified *cis* eQTL for 60% of transcripts (14,807; Table 1) and at least one *trans* eQTL for 56.7% (13,997) transcripts. The number of *trans* eQTL identified per transcript ranged from one (8,092; 32.8% transcripts) to seven (3; 0.01% transcripts).

We next examined eQTL dominance and effect size. Most *cis* eQTL (93.8%; Fig. S1A) were additive (mean for heterozygotes is intermediate and >2 standard errors from both homozygous means – see Methods). In contrast, a substantial proportion of *trans* eQTL were dominant (37.1%), underdominant (9.2%), or overdominant (8.6%). Curiously, *musculus* ^PWD^ alleles were more likely to be dominant among *cis* (473/859; 55.1%) and *trans* eQTL (2,850/4,580; 62.2%). We cannot think of an experimental or biological explanation for this bias. The two categories of eQTL differed in effect size (Fig. S1B). The difference in expression level between genotype classes was larger on average for *cis* eQTL than for *trans* eQTL (t = 72.3 (d.f. = 15931), *P*<2.2×10^−16^). The difference in effect size is also apparent when comparing the peak LOD scores of *cis* (mean = 25.05) and *trans* eQTL (mean = 5.94).

We tested for clustering of *trans* eQTL, which is commonly observed in eQTL analyses [36]–[38]. Some of these ‘*trans* hotspots’ are visible as vertical bands in the eQTL heatmap (Fig. 2). We identified 12 genomic regions significantly enriched for *trans* eQTL using a sliding window analysis (*P*<0.05, permutation test; Table 2). Two adjacent hotspots on chromosome 10 were combined for simplicity in downstream analyses. The most striking pattern was observed for the X chromosome: most of the X was significantly enriched for *trans* eQTL and 8,286 autosomal transcripts (34.6%) had eQTL mapped to the proximal X hotspot (0–42 cM). We discuss the massive effect of the X on gene expression in detail below, and relate this pattern to the known importance of the X in hybrid male sterility.

**Table 2:** *trans* eQTL hotspots.

					Low expression sterile		High expression sterile		
Chr	Position cM	Position Mb^1^	No. *trans* eQTL	“sterile” allele^2^	No. *trans* eQTL	Expression pattern^3^	No. *trans* eQTL	Expression pattern^3^	Sterility QTL overlapping^4^
2	26–38	78.68–119.28	660	D	293	F1.U; MI^B^; ME^C^; PM^A,C,D^	367	F1.O; SO^A,D^; MI^A,D^	TW^D^
3	38–44	86.05–105.42	187	M	107	F1.U; ME^A^; PM^A^	80	F1.O; SO^A,B^; MI^A^	none
5	68–71.50	143.82–148.35	187	D	118	F1.U; ME^A^; PM^A,C,D^	69	F1.O; MI^A,B^; ME^D^	DBT^D^, TAS^D^
6	30–34	89.94–97.3	159	D	110	F1.U; MI^B^; PM^A,C,D^	49	F1.O; MI^A,D^	none
10	4–24*	36.31–78.1	1,328	D	487	F1.U; ME^A^; PM^A,D^	840	F1.O; SO^A,B,D^; MI^A,D^; ME^D^	PBT^D^, TW^D^
11	54–62	78.32–97.18	599	D	332	F1.U; SO^D^; ME^A^; PM^D^	267	F1.O; SO^A,B^; MI^A,D^	none
15	18–38	61.22–88.35	1,551	D	557	F1.U; ME^A^; PM^D^	994	F1.O; SO^A,D^; MI^A,D^	TAS^M^
15	46–50	94.63–97.35	147	D	40	F1.U; SO^D^; ME^A,C^; PM^B,C,D^	107	F1.O; SO^A^; MI^A,B^; ME^B^	none
17	0–16	3.06–38.01	2,435	H	950	F1.U; ME^A,C^; PM^A,C,D^	883	F1.O; SO^A^; MI^A,D^; ME^D^	SD^H^, TW^H^
				M	299	F1.O; F1.U; ME^A^	303	F1.O; F1.U; SO^A^; MI^A,D^	
X	0–42	10.16–101.19	8,286	M	4239	F1.U; ME^A,C^; PM^A,C,D^	4046	F1.O; SO^A,B,D^; MI^A,B,D^; ME^D^	PC1^M^, DBT^M^, ASH^M^, PBT^M^, H/T^M^, SD^M^
X	44–66	106.91–164.43	1,180	D	624	F1.U; PM^A^	556	ME^6^	PC1^M^

1 interpolated.

2 allele with expression pattern consistent with sterility.

3 QTT significantly enriched (hypergeometric test with Bonferroni correction) for transcripts misexpressed in MxD F1s or expressed in a spermatogenic cell type. F1.U - underexpressed in MxD F1s; F1.O – overexpressed in MxD F1s; SO – somatic cell expression; MI – mitotic cell expression; ME – meiotic cell expression; PM – postmeiotic cell expression. Superscript indicates citation for cell types: ^A^ [28]; ^B^ [17]; ^C^ [107]; ^D^ [108].

4 single or MQM, sterile allele is indicated in superscript: D – *domesticus*, M – *musculus*, H- underdominant [1].

* two adjacent regions from 4–16 cM and 16–24 cM combined; sliding window from 14–18 cM is not significantly enriched.

The genomic distribution of eQTL we identified, as well as differences in dominance and effect sizes between *cis* and *trans* eQTL, are broadly consistent with patterns previously described in eQTL studies performed in a variety of (non-hybrid) organisms (*e.g.* humans: [37], [39]; *C. elegans*: [36], [40], [41]; *Arabidopsis*: [32], mice: [42]. This consistency indicates that misexpression and differences in expression level due to altered cell-composition associated with sterility phenotypes were not so severe that they obscured quantitative expression differences between *musculus* ^PWD^ and *domesticus* ^WSB^.

#### *Trans* eQTL hotspots are related to hybrid sterility

Testis eQTL could be related to hybrid sterility or to subspecific differences in gene expression that are independent of hybrid incompatibilities. To distinguish these possibilities, we repeated the eQTL analysis for a subset of F_2_s without strong evidence for hybrid sterility, and compared the results to patterns arising when all individuals were included in the mapping. The number of *cis* eQTL was very similar between samples; we identified 14,501 *cis* eQTL in the fertile sample and 14,807 *cis* eQTL using all individuals. By contrast, patterns of *trans* eQTL showed striking differences between the fertile subset and the full dataset. The number of *trans* eQTL identified (13,652; 7,812 autosomal; 5,839 X) in fertile mice was much lower than in the complete sample (21,946; 12,347 autosomal; 9,599 X). Moreover, clustering of *trans* eQTL was dramatically reduced when only fertile individuals were included (Fig. S2). These results suggest *trans* eQTL, and in particular *trans* hotspots, are related to sterility whereas *cis* eQTL largely reflect subspecific/strain differences in expression. Consequently, expression patterns associated with hotspots – like testis weight, sperm count and other traditional reproductive measures – can be treated as sterility phenotypes.

**Figure 3:**
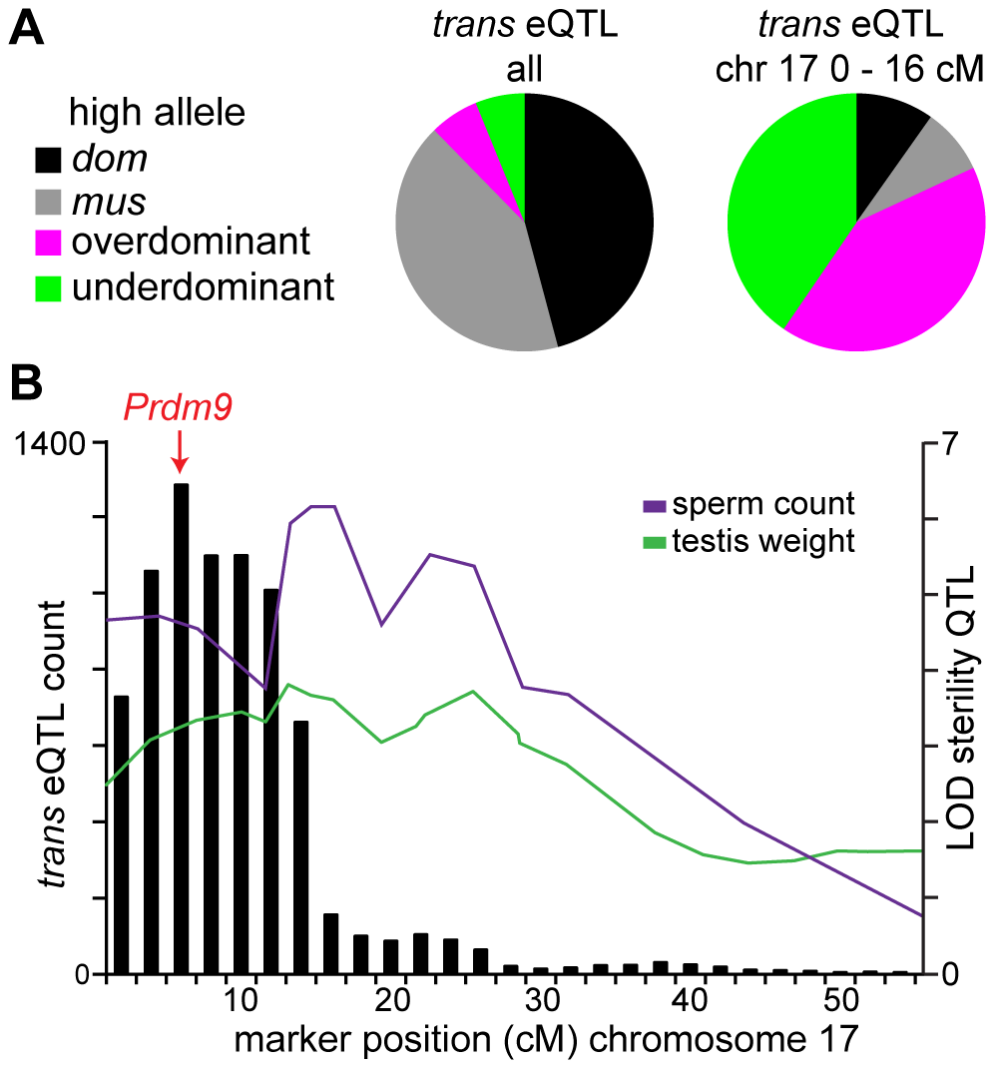
Chromosome 17 hotspot position and effects implicate *Prdm9*. (A) Pie charts showing *trans* eQTL in the chromosome 17 hotspot are largely under- or over-dominant, in contrast to the pattern seen for *trans* eQTL overall. (B) Histogram of *trans* eQTL counts for 4 cM sliding window overlaid with LOD plots for coincident sperm count and testis weight QTL.

To further investigate associations between *trans* hotspots and sterility, we inferred a “sterile allele” for each hotspot as the allele matching the sterile MxD F_1_ pattern and/or showing lower expression of meiotic/postmeiotic transcripts (Table 2). The hotspot on chromosome 17 showed an unusual pattern. A majority of eQTL in the hotspot were over- or underdominant (Fig. 3A) and the heterozygous genotypic class shows evidence for sterility, consistent with the underdominant testis weight QTL at the same position. For additive/dominant eQTL, the *musculus* ^PWD^ genotypic class appeared to be associated with sterility. As we discussed above, both genotypic classes were also associated with overexpression on the X.

The two *trans* hotspots on the X chromosome showed different patterns. The *musculus* ^PWD^ allele at eQTL in the proximal hotspot was associated with the ‘sterile’ expression pattern (Table 2). For example, a substantial proportion of QTT associated with the X hotspots were misexpressed in sterile MxD F_1_s (32.5% X-hotspot QTT vs. 17.3% all autosomal transcripts), with the effect of the *musculus* ^PWD^ allele consistent with the direction of misexpression. In contrast, the *domesticus* ^WSB^ allele was associated with the sterile pattern in the distal hotspot. Correlations between expression levels of QTT and relative right testis weight provided further corroboration for the inferred sterile alleles for the X hotspots. We expected that the sterile allele would cause lower expression of QTT that were positively correlated with testis weight (lower expression with lower testis weight) and higher expression of QTT negatively correlated with testis weight (higher expression with lower testis weight). Most QTT (83.2%) with lower expression caused by the *musculus* ^PWD^ allele at eQTL in the proximal hotspot were positively correlated with testis weight, and most eQTL in the distal hotspot showed the opposite pattern (64.0% negatively correlated). The converse was observed for low-expression *domesticus* ^WSB^ alleles (87.9% QTT in proximal hotspot negatively correlated; 74.0% QTT in distal hotspot positively correlated).

Seven of eleven *trans* hotspots overlapped one or more sterility QTL identified previously in this cross [1] (Table 2; Fig. S2). A total of 99.5 cM was located in a *trans* hotspot and within the 1.5-LOD interval of a sterility QTL (*P* = 0.02, 10,000 permutations of *trans* hotspot positions). For five of seven hotspots overlapping sterility QTL, the sterile allele identified on the basis of expression pattern matched the sterility QTL allele (Table 2), suggesting the underlying causative gene(s) for expression variation and the sterility phenotypes might be shared. One exception is on chromosome 15. The 1.5-LOD interval for a total abnormal sperm QTL overlapped the *trans* hotspot, however the QTL peak (4 cM) was relatively far from the hotspot (18–38 cM; Fig. S2). The second exception was a sperm head-shape QTL (*musculus* ^PWD^ allele sterile) that overlaps the distal-X hotspot (*domesticus* ^WSB^ allele sterile). This region likely harbors sterility QTL from *musculus* ^PWD^ and *domesticus* ^WSB^, providing additional evidence that the role of the X in sterility is complex.

For the present study, we focus on the dramatic patterns of *trans* eQTL and the unexpected association between *trans* hotspots and sterility. We acknowledge that *cis* eQTL may play an important role in hybrid sterility and we anticipate they will be useful in future studies to identify and evaluate candidate sterility genes.

#### Functional annotation of *trans* eQTL hotspots

Characterizing the QTT affected by each hotspot will provide clues about how the underlying genes disrupt fertility. We used DAVID functional annotation [43], [44] to identify classes of genes enriched among QTT with higher or lower expression in individuals with the sterile genotype at each hotspot. Overall, more gene classes were significantly enriched among QTT with higher expression associated with the sterile allele (Table 3). Many of these classes represent basic cell functions including lipid synthesis and metabolism, mitochondrion, and amino acid metabolism (chromosome 11, 15 proximal, 17, X proximal hotspots). Another general pattern was higher expression of classes related to receptors, signaling (*e.g.* transmembrane, glycoprotein, protein kinase-C binding) and specific signaling pathways (PPAR signaling pathway, regulation of MAPKKK cascade).

**Table 3:** Functional annotation of QTT associated with *trans* eQTL hotspots.

Chr	Position cM	Sterile allele	Low expression sterile functional Annotation*	High expression sterile functional Annotation*
**2**	26–36	D		glycoprotein (165); protein kinase C binding (5); *phosphoprotein (165); macrophage (29); cytokine-cytokine receptor interaction (15)*
**3**	38–44	M		glycoprotein (26)
**5**	68–71.50	D		
**6**	30–34	D		
**10**	4–24	D	cytoskeleton (45); microtubule cytoskeleton (22); G protein-coupled olfactory receptor, class II (19)	transmembrane (285)
**11**	54–62	D	chromatin (10)	transmembrane (95); glycoprotein (89); oxidoreductase (26); mitochondrion (25); lipid biosynthetic process (24); fatty acid metabolism (14); butanoate metabolism (14); lysosome (13); NADP (11); arginine and proline metabolism (6); lipid metabolism (5); steroidogenesis (4); intramolecular oxidoreductase activity, transposing C = C bonds (4); *PPAR signaling pathway (6)*
**15**	18–38	D	*plasma membrane (63)*	membrane (326); glycoprotein (256); mitochondrion (118); microsome (51); response to organic substance (44); iron (35); NAD (29); flavoprotein (27); lipid metabolism (26); gland development (25); steroid metabolic process (22); NADP-binding domain (21); gland morphogenesis (14); valine, leucine and isoleucine degradation (12); *oxidation reduction (76); oxidoreductase (68); endoplasmic reticulum (59); cofactor binding (28); NADP (23); heart morphogenesis (14); binding site:substrate via amide nitrogen (8)*
**15**	46–50	D		
**17**	0–16	H	spermatogenesis (36); microtubule organizing center (20); *alternative splicing (308); splice variant (305)*	nucleus (240); ubiquitin conjugation (42); RNA-binding (39); lipid synthesis (23); NAD (21); ATP (21); lipid metabolism (18); melanosome (17); sterol metabolic process (15); *phosphoprotein (460); cytoplasm (184); acetylation (177); endoplasmic reticulum (86); repressor (36); methylation (21); isomerase (18); nucleotide binding (12); P-loop (12)*
		M		oxioreductase (25); fatty acid metabolism (16); steroid biosynthetic process (8); valine, leucine and isoleucine degradation (7); *active site: proton acceptor (26); generation of precursor metabolites and energy (14); NAD (10)*
**X**	0–42	M	sexual reproduction (120); secretory granule (33); fertilization (29); *alternative splicing (930); splice variant (927); acrosomal vesicle (21); flagellum (20); microtubule-based flagellum (14)*	membrane (1016); metal-binding (532); mitochondrion (411); protein transport (408); nucleotide-binding (373); transcription regulation (354); transferase (294); organelle lumen (274); regulation of transferase activity (168); small GTPase mediated signal transduction (161); cell fraction (144); vesicle (137); apoptosis (133); membrane fraction (116); actin-binding (99); hemopoietic or lymphoid organ development (80); lysosome (78); vasculature development (74); cell migration (68); regulation of protein polymerization (65); actin filament-based process (59); basolateral plasma membrane (46); fatty acid metabolism (42); peroxisome (34); SH2 domain (33); flavoprotein (32); regulation of MAPKKK cascade (30); GTP binding (26); valine, leucine and isoleucine degradation (23); histone deacetylase complex (16); T-helper 1 type immune response (8); *phosphoprotein (1501); cytoplasm (699); acetylation (580); intracellular signaling cascade (244); endoplasmic reticulum (187); Golgi apparatus (184); cytosol (153); oxidoreductase (132); topological domain: lumenal (121); endosome (86); nucleolus (84); cell leading edge (44); cell soma (37); melanosome (35); pigment granule (35); colorectal cancer (34); gap junction (34); soluble fraction (33); lamellipodium (27); ruffle (21)*
**X**	44–66	D	glycoprotein (226); kinase (37); cell junction (30); cell adhesion (25); immunoglobulin domain (21); neuromuscular junction (6); *alternative splicing (170); plasma membrane (112); cell membrane (66)*	DNA-binding (57)

* Terms in plain type represent enriched clusters of functionally related genes identified using DAVID functional annotation [43], [44]. For each cluster with at least one annotation term with Benjamini FDR<0.10, the term with the lowest FDR is listed and the number of unique genes in the cluster is in parentheses. Significant annotation terms (FDR<0.10) not assigned to any cluster are listed in italics, and the number of unique genes in parentheses.

As expected, gene classes with clear links to sterility phenotypes were enriched among QTT with lower expression in sterile genotypic classes. The two hotspots coincident with regions known to have major roles in F_1_ sterility showed lower expression of broad categories, including spermatogenesis (chromosome 17), sexual reproduction (X 0–42 cM), and fertilization (X 0–42 cM), as well as classes suggesting more specific sterility-related functions, including microtubule organizing center (chromosome 17) and flagellum/microtubule-associated flagellum (X 0–42 cM). The chromosome-10 hotspot is coincident with an abnormal sperm morphology QTL (proximal bent tail; [1]); lower expression of microtubule genes associated with the sterile *domesticus* ^WSB^ allele is a promising lead for identifying specific disruptions in spermatogenesis.

As discussed above, gene expression measures from whole testis reflect both absolute (per-cell) expression variation and relative expression variation caused by differences in cell-type composition. Genes underlying *trans* hotspots might (1) directly regulate expression of many genes, (2) indirectly affect gene regulation by disrupting an upstream pathway/process, or (3) directly/indirectly cause a change in testis cell composition resulting in altered relative expression of genes from different cell types. We were concerned that many sterility loci might cause similar changes in cell-type composition, in which case annotating hotspots might not be useful for characterizing the function and identify of specific causative genes. If there were a general ‘sterile’ expression pattern we would expect eQTL to be shared across hotspots, particularly for QTT associated with QTL for the same sterility phenotype. In contrast, most QTT with eQTL in hotspots were associated with a single hotspot (8,093/11,904). Variation among QTT associated with different *trans* hotspots and sterility QTL indicates that annotation of these QTT will be informative about functions of individual sterility genes with effects on gene regulation or cell composition. Candidate genes in each hotspot with known roles in male reproduction and/or gene regulation are listed in Table 4.

**Table 4:** Candidate genes in *trans* eQTL hotspots.

Chr	Position Mb	No. Genes^1^	Pseudogenes	non-coding RNAs short/long^2^	No. Coding Genes (with probes)^3^	Probes expressed (total No.)	Differentially expressed parents (WSB/PWD)	Misexpressed MxD F1	*cis* eQTL	Male reproduction/meiosis genes	Gene expression genes
2	78.68–119.28	864	226	48/83	506 (453)	330 (690)	177	31	132	*Bub1b* ^A^; *Cd59b* ^A^; *Fshb* ^A^; *Itgav; Rad51*	*Alx^4^; Bahd1; Cd44; Celf1^A^; Creb3l1; Cry2; Cstf3; Ctnnd1; Ehf; Elf5; Elp^4^; Fmn1; Gm4222; Grem1; Hipk3; Lgr4^A^; Lmo2; Mapk8ip1; Mdk; Meis2; Neurod1; Nr1h3; Pax6; Sfpi1; Ssrp1; Traf6; Wt1^A^; Zfp770*
3	86.05–105.42	547	49	63/29	443 (328)	416 (570)	249	62	183	*Celf3;* * *Creb3l4; Hormad1* **^B^**; *Lmna* ^A^; *Nhlh2* ^A^; *Pygo2* ^A^; *Smcp* ^A^; *Sycp1* ^B^	*2500003M10Rik; 5830417I10Rik; Adar; Arnt; Ash1l; Clk2;* * *Creb3l4; Crtc2; Csde1; Etv3; Gabpb2; Gatad2b; Gon4l; Hdgf; Hipk1; Hist2h4; Ilf2; Iqgap3; Lass2; Mef2d; Mllt11; Mrpl24; Mrpl9; Mrps21; Msto1; Ngf; Nr1h5; Pbxip1; Phgdh; Phtf1; Pias3; Pmf1; Polr3c; Psmd4; Rfx5; Rorc; Rps27; Rrnad1; Tbx15; Trim33; Ttf2; Txnip; Vps72; Zbtb7b; Zfp687*
5	143.82–148.35	162	12	15/16	116 (107)	150 (203)	93	17	66	* *Brca2* ^A^; *Katnal1* ^C^; *Lmtk2* ^A^; *Pms2* ^A^; *Usp42*	*Aimp2; Bhlha15;* * *Brca2^A^; Cdk8; Cdx2; Eif2ak1; Gsx1; Gtf3a; Mtif3; Pdx1; Polr1d; Rasl11a; Rbak; Rnf6; Rpl21; Trrap; Zfp12; Zfp316; Zfp498; Zfp655; Zkscan14; Zkscan5*
6	89.94–97.3	80	8	11/5	54 (50)	63 (89)	36	14	28	* *Mrps25* ^A^; * *Nr2c2* ^A^	*Hdac11; Klf15;* * *Mrps25^A^;* * *Nr2c2^A^; Tmf1; Uba3; Wnt7a^A^; Zxdc*
10	36.31–78.1	436	32	60/26	307 (259)	375 (512)	209	47	177	* *Dnmt3l* ^A^; *Ggt1* ^A^; *Gopc* ^A^; *Herc4;* * *Hsf2* **^A^**; * *Ros1* ^A^; *Sgpl1;* * *Sirt1* **^A^**; *Slc22a16; Tbata*	*Adarb1; Ado; Adora2a;* * *Aire^A^; Arid5b; Ascc1; Asf1a; Atoh7; AW822073; Ccar1; Cdk1;* * *Dnmt3l^A^; Egr2; Foxo3; Fyn;* * *Gja1^A^; Gm4981; Gtf3c6; Hace1; Hdac2;* * *Hsf2^A^; Jmjd1c; Lin28b; Neurog3; Nr2e1; Pcbd1;* * *Prdm1^A^; Prmt2; Rfx6;* * *Ros1^A^; Scml4; Sim1;* * *Sirt1^A^; Smarcb1; Tet1; Tfam; Vgll2; Zbtb24; Zfa*
11	78.32–97.18	461	73	56/70	254 (241)	334 (471)	177	41	149	*Ggnbp2;* * *Hils1; Mycbpap; Ppm1d* ^A^; *Rad51c* **^A^**; *Sept4* ^A^; *Spag9* ^A^; *Spata20*; *Tex14* ^A^; *Tubd1*	*1110002N22Rik; 1500016L03Rik; Aatf; Cbx1; Ccl5; Cdk5r1; Col1a1; Crlf3; Dlx3; Dlx4; Fam58b;* * *Hils1; Hlf; Hnf1b; Hoxb1; Hoxb2; Hoxb3; Hoxb4; Hoxb5; Hoxb6; Hoxb7; Hoxb8; Hoxb9; Hoxb13; Hsf5; Lhx1; Mbtd1; Med13; Mrm1; Mrpl10; Mrpl27; Mrps23; Myst2; Nfe2l1; Ngfr; Nlk; Nme1; Phb; Skap1; Sp6; Supt4h1; Suz12; Tada2a; Tbx2; Tbx4; Tbx21; Utp6; Zfp652*
15	61.22–88.35	431	19	52/18	335 (296)	428 (516)	255	64	170	*Bik; Dmc1* **^A^**; * *Hsf1* **^A^**; *Mei1* **^A^**; *Smc1b* **^A^**; * *Srebf2; Sstr3; Tssk5;* * *Zfp41*	* *Atf4^A^; Cbx6; Cbx7; Cby1; Chadl; Csdc2; Ddx17; Efcab6; Eif2c2; Ep300; Exosc4; Foxh1; Gm4825; Gm5218; Gm8444; Gm10923;* * *Hsf1^A^; Khdrbs3; L3mbtl2; Maf1; Mafa; Maff; Mkl1; Myc; Pdgfb; Phf5a; Polr2f; Polr3h; Ppara; Puf60; Rbfox2; Rpl3; Rpl8; Scrt1; Scx; Sox10;* * *Srebf2; Tef; Tob2; Wnt7b; Xrcc6; Zfat; Zfp7;* * *Zfp41; Zfp251; Zfp647; Zfp707*
15	94.63–97.35	20	1	5/1	13 (11)	24 (36)	14	5	9		*Arid2; Dbx2*
17	3.06–38.01	1021	179	72/64	733 (541)	634 (850)	351	89	312	*4930528F23Rik; Bag6^A^;* * *Bak1;* * *Ehmt2; Ggnbp1; Hspa1l; Mas1; Msh5^A^; Pacrg^A^;* * *Prdm9^B,D^; Prss21; Rnf151; Rsph1; Slc26a8;* * *Sox8^A^;* * *Tbp; Tcp11; Tekt4^A^*	*2210404O09Rik; 3110052M02Rik; 4921501E09Rik; Abcg1; Aif1; Atf6b; Axin1;* * *Bak1; Cdkn1a; Chd1; Cryaa; Daxx; E4f1;* * *Ehmt2; Fam120b; Gm6712; Gm8225; Gtf2h4; Gtf2h5; Haghl; Hmga1; Lmf1; Ltb; Mapk13; Mapk14; Mapk8ip3; Mrpl18; Mrpl28; Mrps18b; Narfl; Nkx2-5; Notch3; Ntn3; Pbx2; Pdpk1; Phf1; Phf10; Pknox1; Pou5f1; Ppard; Ppp1r10;* * *Prdm9^B,D^; Qk; Rab26; Rdbp; Rgmb; Ring1; Rpl3l; Rpl10a; Rps2; Rps18; Rps28; Rps6ka2;* * *Rxrb^A^; Scaf8;* * *Sox8^A^; Spdef; Srpk1; Stub1; T; Taf11;* * *Tbp; Tceb2; Tead3; Tfb1m; Tnf; Traf7; Ube2i; Zbtb9; Zbtb22; Zfp13; Zfp40; Zfp51; Zfp52; Zfp53; Zfp54; Zfp57; Zfp81; Zfp97; Zfp101; Zfp160; Zfp213; Zfp229; Zfp414; Zfp422-rs1; Zfp472; Zfp523; Zfp563; Zfp677; Zfp758; Zfp760; Zfp763; Zfp799; Zfp811; Zfp820; Zfp870; Zfp871; Zfp942; Zfp943; Zfp944; Zfp945; Zfp946; Zfp947; Zfp948; Zfp952; Zfp955a; Zfp955b; Zfp960; Znrd1; Zscan10*
X	10.16–101.19	1610	720	176/75	819 (421)	484 (761)	260	114	242	*Akap4^A^; Akap14; C430049B03Rik; Ccnb3; Cetn2; Fmr1^A^; Gm2825; Hprt;* * *Nr0b1^A^; Pcyt1b^A^;* * *Sox3^A^; Stag2; Tex11^A^;* * *Zfx^A^*	*A230072C01Rik; Aff2; Agtr2; Akap17b;* * *Ar^A^; Arx; Atp1b4; Avpr2; Bcor; Bcorl1; Cdx4; Cited1; Dkc1; Dmd; Dmrtc1a; Eda; Elf4; Elk1; Foxo4; Foxp3; Gata1; Gm4987; Gm5751; Gm6592; Gm14459; Gm14543; Gm14647; Gm20464; Gspt2; Hcfc1; Hdac6; Hdac8; Hsf3; Htatsf1; Igsf1; Ikbkg; Il2rg; Irak1; Maged1; Mamld1; Mbnl3; Mcts1; Mecp2; Med12; Med14; Mycs; Ndp; Nkap; Nkrf; Nono;* * *Nr0b1^A^; Phf6; Pqbp1; Rbm3; Rbmx; Rhox1; Rhox2a; Rhox2b; Rhox2c; Rhox2d; Rhox2e; Rhox2f; Rhox2g; Rhox2h; Rhox3a; Rhox3c; Rhox3e; Rhox3f; Rhox3h; Rhox4a; Rhox4b; Rhox4c; Rhox4d; Rhox4e; Rhox4f; Rhox4g; Rhox5; Rhox6; Rhox7; Rhox8; Rhox9; Rhox10; Rhox11; Rhox12; Rhox13; Rlim; Rpl10; Rpl39; Rps4x; Smarca1;* * *Sox3^A^; Ssx9; Ssxa1; Ssxb1; Ssxb2; Ssxb3; Ssxb5; Ssxb6; Ssxb8; Ssxb9; Ssxb10; Suv39h1; Taf1; Tbl1x; Tfe3; Vgll1; Zbtb33; Zcchc12; Zfp92; Zfp182; Zfp275; Zfp280c; Zfp300; Zfp449;* * *Zfx^A^; Zic3; Zxdb*
X	106.91–164.43	873	401	108/52	392 (220)	237 (384)	134	64	125	*Gpr64* ^A^; *Piga* ^A^; *Smc1a;* * *Taf7l* **^A^**	*Btk; Dach2; Esx1; Foxr2; Hdx; Kdm5c; Klf8; Morf4l2; Phf8; Plp1; Pou3f4; Rbbp7; Ripply1; Rpl36a; Scml2;* * *Taf7l^A^; Tceal1; Tceal5; Tceal7; Tceal8; Tceanc; Tgif2lx1; Tgif2lx2; Tmsb4x; Trappc2; Trmt2b; Tsc22d3; Tspyl2; Txlng; Yy2; Zfp711*

1 Ensembl gene IDs.

2 short (miRNAs, snRNAs, snoRNAs)/long (antisense and linc RNAs).

3 genes with probes on the Agilent Whole Mouse Genome array are listed in parentheses.

* genes with reproductive and gene expression functions.

Genes with characterized effects of null mutants on male fertility: ^A^ [17]; ^B^ [105]; ^C^ [109]; ^D^ [27].

### Genetic interactions identified by conditional mapping of eQTL

The Dobzhansky-Muller model predicts that each hybrid sterility locus will have one or more interaction partners. Mapping of genetic interactions generally requires sample sizes larger than the 305 F_2_s analyzed here. To increase power, we treated *trans* eQTL hotspots as candidate hybrid sterility loci and searched for interactions involving them. We performed conditional mapping of eQTL, using genotypes at candidate loci one at a time as covariates. Genotype covariates included the marker closest to the peak of each of the nine autosomal *trans* eQTL hotspots, and five markers in the X chromosome *trans* hotspots (Table 5). For each covariate, mapping was performed twice, including an additive effect or both an additive and interactive effect; eQTL from the full model that showed a significant increase in LOD score over the additive model were classified as significant interaction eQTL.

**Table 5:** Conditional mapping results by covariate.

Covariate Chr	Covariate marker	Covariate Position cM	Covariate Position Mb	Interaction eQTL	No. Interaction Hotspots	No. Chrs. Hotspots^1^	% covariate marginal effect^2^	% peak marginal effect^3^	% reciprocal interaction^4^
2	NES09108608	28.8	92.00	2864	21	14	4.7	10.2	6.0
3	NES13927401	40.7	96.20	1665	10	8	1.9	18.2	4.0
5	NES10364112	71.5	148.40	3586	21	14	1.6	14.6	5.4
6	NES11922718	32.6	93.30	1635	15	8	1.9	11.6	10.8
10	NES16893219	6.2	40.50	1032	9	7	2.2	12.3	10.1
11	NES14174531	18.6	65.10	1025	14	10	3.7	10.3	12.0
15	NES08577121	59.4	91.70	1273	10	6	3.8	11.3	5.8
15	NES17019164	21.8	68.70	1050	8	4	2.6	11.7	6.6
17	NES16574315	13.3	30.00	3525	10	9	17.7	36.2	36.0
X	NES12384176	15.0	58.30	6493	14	9	37.8	15.5	33.1
X	NES09680036	24.0	84.00	2009	15	9	15.9	11.3	47.6
X	NES09660234	33.4	97.40	1401	10	8	5.6	9.0	41.3
X	NES09767342	52.2	131.80	2209	15	9	9.9	8.7	15.4
X	NES11023996	63.7	162.90	1171	10	7	7.2	11.3	24.3

1 Number chromosomes harboring interaction eQTL hotspots.

2–3 Percentage of interaction eQTL for which there was a significant eQTL at the ^2^covariate position or ^3^peak position in the original eQTL mapping analysis (no covariate).

4 Percentage of interaction eQTL for which there was a significant interaction eQTL with the covariate and peak positions reversed.

**Figure 4:**
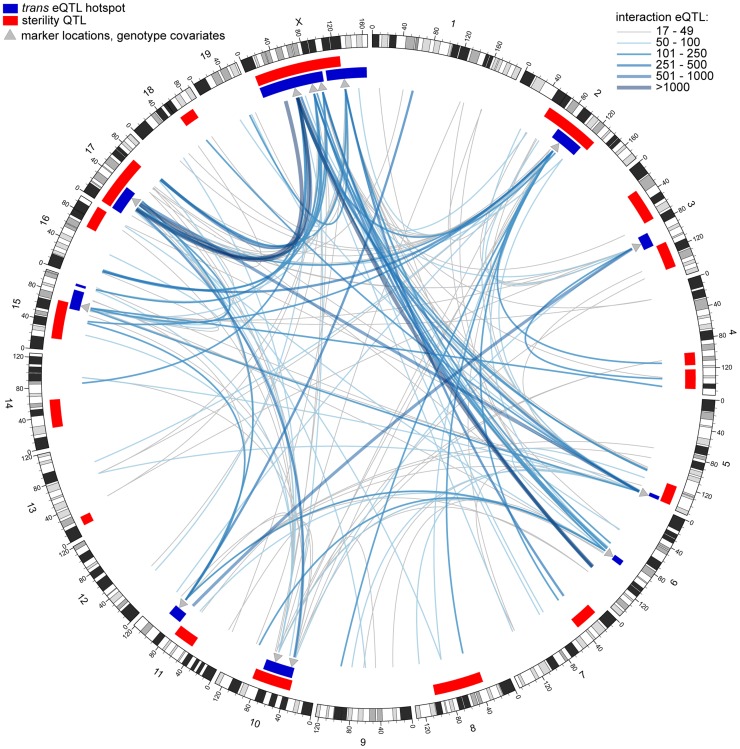
Genetic interactions revealed by conditional mapping. Genome plot generated using circos software [106]. Each line represents an interaction eQTL hotspot; color and thickness indicate number of eQTL. Red rectangles indicate sterility QTL positions and dark blue rectangles indicate *trans* eQTL hotspots (original mapping). Grey triangles indicate positions of marker genotypes used as covariates in conditional mapping.

Clustering of interaction eQTL identified by conditional mapping was even more pronounced than clustering of *trans* eQTL in the initial (no covariate) eQTL analysis (Fig. S3). We identified ‘interaction hotspots’ using significance thresholds from permutation for each genotype covariate. Integrating results from the conditional mapping analyses reveals a complex epistatic network showing several general patterns (Fig. 4). The large number of interactions involving the X is consistent with its substantive effect on expression pattern and sterility phenotypes. There are many interactions between loci in *trans* hotspots, and between *trans* hotspots and sterility QTL, suggesting that some incompatibilities contribute to multiple phenotypes. Overall, a large proportion of interactions are associated with sterility loci. It is important to note that many interactions may be associated with variation in gene expression unrelated to hybrid sterility.

The interactions we identified include X-autosome pairs previously associated with hybrid sterility. We identified interaction hotspots in the proximal region of chromosome 17, which encompasses *Prdm9*, from conditional mapping using all X-linked genotype covariates; conversely, mapping conditional on Chr17@13 cM identified a hotspot on the proximal X (Fig. S4). Previous mapping of sterility phenotypes conditional on X genotypes revealed interactions between the X and six autosomal regions on four chromosomes (3, 5, 7, 10), contributing to five sperm morphology phenotypes [1]. We found interaction hotspots involving at least one X-linked covariate overlapping each of these autosomal regions.

**Figure 5:**
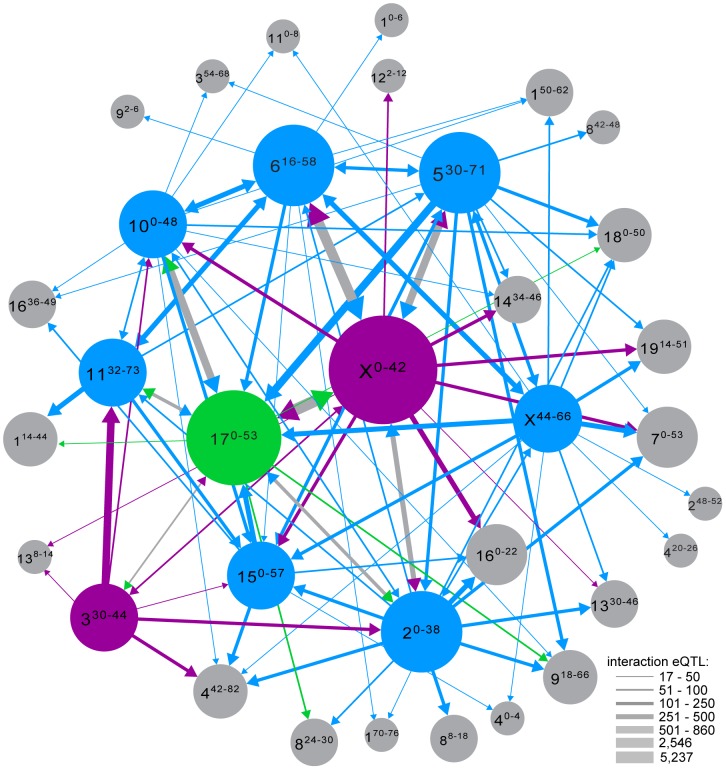
Interaction network. Interaction eQTL hotspots identified with different genotype covariates are shown as single nodes if the distance between regions was <12.8 Mb (average distance between genotyping markers). Nodes are labeled with chromosome, and position (cM) in superscript. Nodes with *musculus* ^PWD^ sterile alleles are magenta, *domesticus* ^WSB^ sterile alleles in blue and sterile heterozygous genotypes in green. Edge weight indicates the number of interaction eQTL. Node size is proportional to total number of interactions. Edge color matches sterile allele at marker used as covariate and arrow points to node of peak position. Edges with two arrowheads indicate reciprocal covariate/peak interactions between nodes; if sterile alleles differ, edge is gray and arrowheads indicate sterile allele at opposite node.

Each *trans* hotspot identified in the original analysis overlapped at least one interaction hotspot mapped with an autosomal covariate, indicating autosome-autosome interactions contribute substantially to expression variation. All of these interactions are novel. Interactions between regions with sterile alleles from the same subspecies are prevalent (Fig. 5), suggesting incompatibilities involving more than two loci are common.

Conditional mapping revealed additional associations between gene expression variation and sterility. Some sterility QTL that did not overlap a *trans* hotspot identified in the original analysis showed evidence for interaction with one or more hotspot regions (Fig. S4). We also found interactions with sterility QTL for each of the *trans* hotspots that do not overlap sterility QTL. The relative contribution of loci to expression variation with detectable marginal effects versus eQTL identified only when incorporating interactions varied (Table 5). The structure of the interaction network provides additional support for the important roles of chromosomes X and 17, the major players in F_1_ sterility (Figs. 4; 5). By contrast, the chromosome 6 region plays a prominent role in the interaction network (Fig. 5), which was unanticipated on the basis of relatively modest enrichment of eQTL in the *trans* hotspot and the lack of sterility phenotype QTL on chromosome 6.

We identified several novel loci that interact with multiple *trans* hotspots but did not have previous evidence for involvement in sterility (Fig. S4). Regions on chromosomes 7 (50–52 cM; 122.63–125.77 Mb), 13 (32–36 cM; 68.47–75.96 Mb), 14 (40–44 cM; 87.59–97.00 Mb) and 16 (0–4 cM; 11.20–20.02 Mb) had overlapping interaction hotspots identified by mapping with genotype covariates from *trans* hotspots on at least three chromosomes. These results indicate that some loci in the interaction network have marginal effects undetectable using single-QTL models and permutation thresholds.

## Discussion

The Dobzhansky-Muller model of reproductive isolation has been well accepted for decades but relatively few incompatible loci and even fewer interactions are known. Due to the central role of negative epistasis in hybrid defects, disruptions in gene networks are likely to be common in hybrids [45]–[47]. Inspired by recent ‘systems genetics’ studies that integrate phenotype, genotype, and gene expression data to reconstruct gene networks and infer relationships between perturbations in networks and deleterious traits [48], [49], we mapped expression traits in an F_2_ cross between house mouse subspecies. We combined expression-mapping results with knowledge of QTL for sterility phenotypes in the same cross to identify altered expression patterns reflecting disruptions in networks causing sterility.

### The role of gene regulation differences in hybrid sterility

The importance of evolutionary changes in transcriptional regulation for adaptation has long been recognized [e.g. 50]–[53]. Recent studies of gene expression in hybrids suggest regulatory evolution may also be an important cause of reproductive isolation between diverging populations. Misexpression has been reported in hybrids from many animal and plant taxa including *Drosophila* [8], [12], [54], mice [9]–[11], [55], African clawed frogs [13], [56], whitefish [57], copepods [58], maize [59], ragwort [60] and *Arabidopsis* [61]. Furthermore, several known hybrid incompatibility genes affect transcription of other genes, including *OdsH* [12] and the mouse sterility gene *Prdm9* [27]. Our expression data from F_1_ and F_2_ hybrids show male sterility is associated with major alterations in genome-wide expression patterns. Clustering of *trans* eQTL is much less pronounced when mapping is restricted to fertile mice (Fig. S2), indicating *trans* hotspots in particular are associated with sterility. Each of the *trans* hotspots we identified overlaps a sterility QTL and/or interacts with at least one region containing a sterility QTL. One interpretation of this pattern is that divergent alleles with major effects on expression patterns are likely to cause hybrid incompatibilities. *Trans* regulators of gene expression must coordinate properly with *cis* regulators and other *trans* factors. The number and broad genomic distribution of regulated genes and co-factors provide many potential opportunities for incompatible interactions resulting in deleterious phenotypes in hybrids. Misexpression of a gene could result from a change in the set of positive or negative regulatory factors, or a mismatch in the spatiotemporal availability of these factors and the timing of expression. This hypothesis suggests genes in interacting regions with large *cis* eQTL and/or major alterations in spatiotemporal expression pattern between subspecies should be prioritized as candidates.

### The role of the X chromosome in hybrid male sterility

Numerous studies of F_1_ hybrid sterility and evidence for reduced gene flow in hybrid zones have shown that the X chromosome plays a central role in hybrid male sterility in house mice [5], [62]–[65]. Our expression mapping results in F_2_s show that the X has a massive effect on testis gene expression, providing support for an important role of the X beyond the F_1_ generation. Most of the X chromosome is significantly enriched for QTL affecting expression of autosomal genes.

The *musculus* ^PWD^ allele in the proximal X hotspot (10.16 Mb–101.19 Mb) has effects on expression suggestive of sterility (Table 2), consistent with the well-documented role of the *musculus* X in F_1_ sterility. This region harbors the largest-effect QTL identified for testis weight, sperm count, abnormal sperm head morphology, and number of offspring in X introgression experiments [25], [66]. Genes with functions related to fertility (sexual reproduction, fertilization, flagellum) were enriched among the QTT with low expression caused by the *musculus* ^PWD^ allele (Table 3).

By contrast, the distal X hotspot shows little similarity to patterns observed in sterile F_1_ males. The distal hotspot overlaps several sterility QTL identified in X*^musculus^* introgression experiments (Supp. Table S2), but the *domesticus* ^WSB^ allele at hotspot eQTL is associated with the sterile expression pattern. These results reveal the presence of at least one novel locus on the X contributing to expression variation and potentially F_2_ sterility (Tables 2, S2). Fertility of DxM F_1_s, which carry the *domesticus* ^WSB^ X, and lack of enrichment of the distal hotspot QTT for transcripts misexpressed in F_1_s, indicate this locus interacts with one or more recessive *musculus* ^PWD^ autosomal loci. DNA-binding genes are enriched among QTT with higher expression, raising the possibility that the distal locus controls expression of regulatory genes, and the role in sterility is indirect.

Variation within the *trans* hotspots on the X suggests each may harbor more than one sterility gene. The number of eQTL mapped, and the proportions of QTT with sterility-related characteristics, varied within the proximal and distal hotspots (Table S2). Furthermore, comparison of conditional mapping results using different markers on the X as covariates reveals differences in interaction patterns (Fig. S5).

#### The mechanism of sterility caused by the proximal X*^musculus^*

Several mechanisms have been proposed for hybrid defects caused by the X. In each case, de-repression of X-linked genes normally transcriptionally silenced during and after meiosis is implicated, but the proposed cause and timing of de-repression differ.

Expression of X- and Y-linked genes is suppressed during meiosis in mice [29], [30]. Meiotic sex chromatin inactivation (MSCI) is essential for spermatogenesis. Mouse models with mutations in MSCI genes show meiotic arrest during the pachytene stage [30]. Overexpression of X-linked genes has been documented in F_1_ studies involving multiple strains [10], [26]. Good and colleagues [10] proposed that overexpression might be related to disrupted MSCI. Measurement of a subset of the overexpressed genes in enriched populations of different testis cell types showed that overexpression was first observed in primary spermatocytes, consistent with the onset of MSCI [31].

The dramatic overexpression of the X observed in F_1_ and F_2_ hybrids documented here is consistent with failed MSCI. We identified QTL on chromosomes 17 and X for the number of overexpressed X-linked transcripts in an individual. The peak of the QTL on chromosome 17 is coincident with the sterility gene *Prdm9* and overexpression is caused by heterozygosity at this locus, consistent with maximal sterility effects in individuals heterozygous at *Prdm9* [67]. The QTL on the X overlaps the *musculus* X region with the strongest overexpression effects in introgression lines [68]. The peak in number of *trans* eQTL we mapped in F_2_s is coincident with the X-overexpression QTL, suggesting effects on expression of autosomal genes are related to overexpression of the X.

A recent investigation of sterility mechanisms in F_1_ hybrids harboring the sterile allele of *Prdm9* documented extensive asynapsis during meiosis, and subsequent meiotic checkpoint failure and arrest [26]. Analysis of intersubspecific chromosome substitution strains showed that heterospecific chromosome pairing was a preexisting condition of asynapsis. *Prdm9* heterozygosity and a *musculus* X were necessary but not sufficient for the full meiotic arrest phenotype. The authors propose that interaction between these loci regulates the stability of pairing between heterospecific chromosomes. Expression of several sex-linked genes during meiosis in these F_1_ hybrids indicates MSCI is disrupted. The authors suggest MSCI failure is a consequence of asynapsis.

Asynapsis of heterospecific chromosomes is unlikely to cause failure of MSCI in F_2_s. Chromosome pairs in F_2_s harbor both conspecific and heterospecific regions, which should enable synapsis in most cases because only one crossover per chromosome arm is required to maintain pairing [69]. Moreover, complete meiotic arrest was rarely observed in F_2_s [1], and the most severe arrest phenotype observed in *musculus* ^PWD^×*domesticus* ^B6^ F_1_s was not seen in our MxD F_1_s. Taken together, results of sterility phenotype and expression mapping in F_2_s suggest the *Prdm9*-X interaction contributes to sterility through mechanisms beyond asynapsis.

Many sex-linked genes remain silenced into spermiogenesis. Disruptions in postmeiotic sex chromosome repression (PSCR) have also been associated with male sterility in mice. Derepression is caused by unbalanced copy numbers of multicopy X- and Y-linked regulatory genes [70], [71]. Expansion and increased copy number variation of some of these genes was observed in mice from a natural hybrid zone [72], suggesting unequal recombination in hybrids might exacerbate effects of copy-number imbalance on PSCR.

In contrast to previous F_1_ studies [10], [31], we did not find evidence for disrupted PSCR: there was no general pattern of overexpression of postmeiotic genes in F_1_ or F_2_ hybrids (Table S1). Decoupling of MSCI and PSCR suggests failed PSCR is not a necessary downstream consequence of disrupted MSCI [31]. Lack of disrupted PSCR in F_2_s is not surprising if *Slx/Sly* imbalance is the major cause because there is not a mismatch between subspecies in the critical regions; most (293/305) F_2_s in this study have a *musculus* ^PWD^ Y (because nearly all MxD F_1_s were sterile) and overexpression/sterility is associated with the *musculus* ^PWD^ allele in the proximal X, which overlaps the positions of *Slx/Slxl*. Lack of mismatch does not explain lack of PSCR in sterile MxD F_1_s, however, suggesting there is polymorphism for alleles causing copy number imbalance. QTL for abnormal sperm morphology, similar to phenotypes reported for *Sly*-deficient males [70], were identified on the X [1], indicating the X harbors multiple loci affecting post-meiotic spermiogenesis.

**Figure 6:**
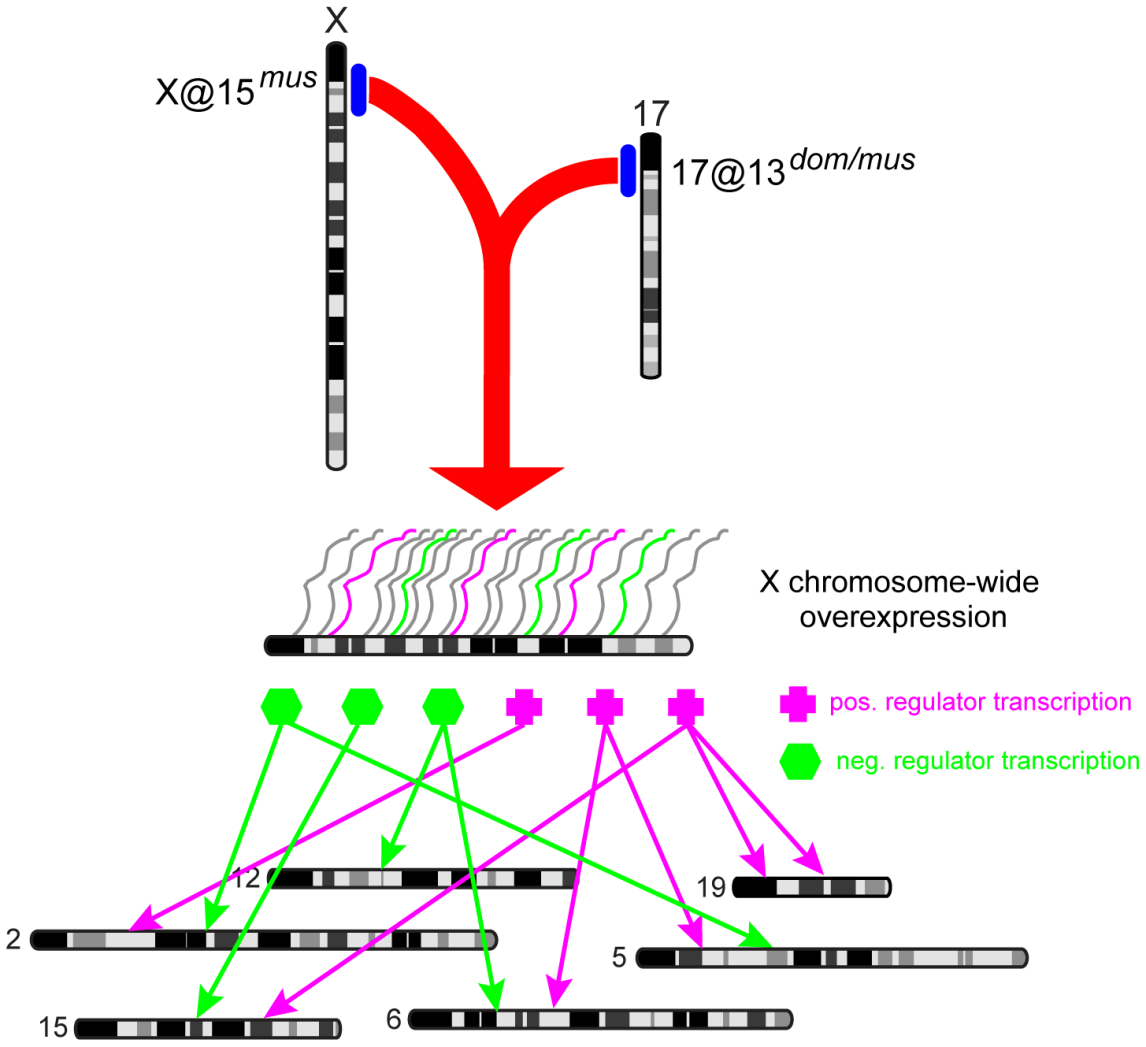
Model of genome-wide expression effects caused by X-17 interaction. A *musculus* ^PWD^ allele on the X @15 cM interacts with heterozygous 17@13 cM (likely *Prdm9*) to cause overexpression of the X chromosome during meiosis. X-linked transcriptional regulation genes, which are usually silenced by MSCI, affect expression of autosomal genes.

We propose that an interaction between loci on chromosome 17@13.3 cM (likely *Prdm9*) and X@15 cM disrupts MSCI, either directly or indirectly. De-repression has a cascading effect on meiotic expression of autosomal genes due to activity of X-linked transcriptional activators/suppressors at stages where they are normally silenced (Fig. 6). This model explains the co-localization of the X-overexpression QTL on the X with thousands of *trans* eQTL affecting expression of autosomal genes.

### Chromosome-17 hotspot reveals novel insights about the role of *Prdm9* in sterility

We identified a region on chromosome 17 with major effects on gene expression. Several lines of evidence implicate the known sterility gene *Prdm9* as the underlying causative gene. First, the QTL for overexpression of X-linked transcripts (18.46 Mb) and the peak in number of *trans* eQTL within the chromosome-17 hotspot (14.69 Mb) are near Prdm9 (15.68 Mb; Fig. 3B). Second, eQTL in the chromosome-17 hotspot largely show under- or overdominant effects, in contrast to *trans* eQTL elsewhere in the genome, which are mostly additive or dominant (Fig. 3A). This pattern is consistent with results from F_1_ crosses showing the most severe sterility phenotypes occur in males heterozygous at *Prdm9* [67]. Finally, we find evidence for interactions between the chromosome-17 region and a *musculus* ^PWD^ allele on the proximal X chromosome, consistent with F_1_ studies [4].

If *Prdm9* is the causative gene, our eQTL results provide novel insights into its role in hybrid sterility and gene regulation. In addition to the known interaction with the X chromosome, we find evidence for interaction with each autosomal locus used as a mapping covariate (Figs. S4; 5). The large number of interacting loci suggests that the DNA-binding function of *Prdm9*, which regulates recombination hotspots globally [73], [74], might be directly related to its role in sterility. Each *Prdm9*-binding site represents a potential incompatibility partner. Alternatively, disrupted regulation caused by *Prdm9* might have cascading effects resulting in altered expression genome-wide.

Although *Prdm9* is predicted to have broad regulatory effects, previous evidence for effects on expression levels was limited to a small set of genes directly regulated by *Prdm9* [27]. The combination of eQTL in the chromosome-17 hotspot (without covariates; Table 2) and eQTL dependent on interactions with eight autosomes and the X chromosome (Table 5) identifies 5,467 unique transcripts directly or indirectly affected by the region encompassing *Prdm9*.

Chromosome 17 harbors a second, more distal sterility locus, *Hstw^s^*, from *musculus* [18]. *Hstw^s^* is necessary, in addition to the sterile *Prdm9^domesticus^* allele and the *musculus* X, to observe complete meiotic arrest, the most severe F_1_ phenotype [67]. We identified interactions between both the *Prdm9* region and a distal chromosome 17 region with chromosomes 2, 5, 10, and X (Fig. S4), suggesting loci on those chromosomes may be involved in the *Prdm9- Hstw^s^* incompatibility.

### Candidate hybrid sterility genes

Overlap of sterility QTL with *trans* hotspots and/or interaction hotspots can refine estimates of the QTL position in some cases. For example, the *trans* hotspot on chromosome 17 is smaller than the coincident QTL for sperm count and testis weight (Fig. 3B). Moreover, the peak in number of *trans* eQTL is at the position closest to *Prdm9*. Chromosomes 5 and 10 are cases where *trans* eQTL and interaction eQTL patterns appear particularly useful in narrowing lists of candidate genes (Fig. S2)

Functional annotation of QTT identifies affected pathways and processes associated with some hotspots, and provide clues about the mechanisms underlying sterility. Chromatin-related genes were overrepresented among QTT with lower expression associated with the sterile *domesticus* ^WSB^ allele at the chromosome 11 hotspot (Table 3). Mouse knockout models for two additional genes with eQTL in this region have spermatogenesis defects that might be related to chromatin; males with null alleles at the transcription factor *Crem* (cAMP responsive element modulator) showed defective spermiogenesis with aberrant post-meiotic gene expression [75]. *Lmna* (lamin A) knockouts have severely impaired spermatogenesis associated with failed chromosomal synapsis [76]. These patterns suggest prioritizing genes in the chromosome 11 hotspot with related functions. For example, 42 genes are involved in transcriptional regulation (Table 4). One of these genes (*Hils1*) is involved in chromatin remodeling during spermatogenesis and has evolved rapidly within rodents [77]. Males with hypomorphic *Rad51c* alleles are infertile due to arrest of spermatogenesis in early meiotic prophase I related to failed double-strand break repair by recombination [78].

Interactions between novel loci and better-characterized regions point to some promising candidates. For example, the chromosome-10 hotspot interacts with the proximal X and the chromosome-17 region containing *Prdm9*, the two loci with the most dramatic effects on expression. A gene within the chromosome-10 hotspot, *Dnmt3l* (DNA methyltransferase 3-like), plays a key role in epigenetic programming during spermatogenesis. Males carrying null alleles at *Dnmt3l* show phenotypes similar to those documented in F_1_s associated with the X-17 interaction, including hypogonadism, asynapsis during meiosis, abnormal formation of the sex body, and deregulation of X-linked and autosomal genes [79]–[82]. *Dnmt3l* does not have methyltransferase activity but shows sequence similarity to *Dnmt3a* and *Dnmt3b*, with which it interacts to promote *de novo* DNA methylation [83]. Misexpression of *Dnmt3a* was reported previously in sterile F_1_ hybrids [10]. *Prdm9* is a histone methyltransferase; while speculative, an interaction between *Dnmt3l* and *Prdm9* is a promising lead. *Dnmt3l* is essential for several epigenetic processes occurring at different stages of spermatogenesis, including paternal imprinting, transcriptional regulation, chromatin morphogenesis through meiosis, and the histone-protamine transition during spermiogenesis. Interestingly, *Dnmt3l* interacts with heterochromatin [80], similar to the *Drosophila* sterility gene *OdsH* [84].

Conditional mapping revealed several genomic regions involved in the interaction network that did not have previous evidence for involvement in sterility or expression (Fig. 5), indicating this mapping approach can uncover incompatibility loci without detectable marginal effects. Some of these interaction loci are very small, containing few enough genes that targeted functional evaluation would be feasible. For example interaction hotspots mapped with covariates on chromosomes 2, 5, and X overlap on distal chromosome 7 (Fig. S4). This region spans 3.1 Mb, encompassing 14 characterized RefSeq genes.

We focused here on genome-wide patterns. Detailed characterization of individual loci, and analysis of gene co-expression networks including all related QTT, will yield additional information useful in pinpointing the disrupted pathways causing sterility and prioritizing candidates.

### Implications for evolution of reproductive barriers

The rate of accumulation of Dobzhansky-Muller incompatibilities and the evolution of reproductive barriers between incipient species depend on the genetic architecture of isolating traits. Theoretical models of DMI evolution assume that incompatibilities act independently on barrier traits [85], [86]. The complex pattern of interactions we report here violates this assumption: some sterility loci are involved in multiple incompatibilities. This aspect of the network we characterized is most consistent with branched developmental pathways [45] and gene networks models [47]. Theory that incorporates this non-independence as well as other biological characteristics of incompatibilities should continue to be pursued [45]–[47], [87].

Network characteristics are also key determinants in accurate modeling of gene-flow dynamics in zones of hybridization. Non-independence of incompatibilities due to interactions of sterility loci with multiple partners is likely to result in stronger selection and slower introgression at those loci because sterility phenotypes are expressed on a variety of genomic backgrounds. Future cline theory should incorporate epistasis with multiple partner loci.

### A network-centered approach to speciation genetics

Remarkable progress in understanding the genetic basis of speciation has emerged from identification of a growing list of hybrid incompatibility genes [6] over the past 20 years. However, identification and functional characterization of hybrid incompatibility genes is feasible in only a few model organisms, and tremendous effort, time and resources are needed to identify a single gene. If this gene-by-gene approach continues as the standard in speciation genetics, it will be a long time before the number of genes and interactions identified is sufficient to reveal generalities of the speciation process. Moreover, general features of incompatibility networks, including the number and dominance of loci, types of interactions, and possibly particular developmental/regulatory pathways, are more likely to be shared among taxa than are specific incompatibility genes.

The house mouse features a rich set of sophisticated genetic tools and resources, which facilitates collection of reliable genome-scale data and ultimately will enable functional characterization of candidate incompatibility genes. Although identification and characterization of reproductive barrier genes is not feasible in most species, the integrated mapping approach we employed is applicable in a broad range of organisms. For species pairs that can be crossed in the laboratory, a similar F_2_ intercross can be performed and sterility or inviability phenotypes can be measured. Informative marker discovery is straight-forward and relatively low cost using RADseq [88], and RNAseq or custom microarrays can be used to collect expression data from species without commercially available platforms. Functional annotation and nomination of candidate processes/pathways is possible if a genome sequence of the focal species or even a relatively distantly related taxon is available [89]. Even in species with very limited available gene annotation, the number of incompatibility loci and the nature of interactions between them can be estimated. Consequently, we suggest that network-centered approaches are powerful and have promise to substantially advance understanding of speciation.

## Materials and Methods

### Ethics statement

Mice were maintained at the University of Wisconsin School of Medicine and Public Health mouse facility according to animal care protocols approved by the University of Wisconsin Animal Care and Use Committee.

### Sterility phenotype data and tissue collection

Reciprocal crosses of wild-derived inbred strains of *M. m. domesticus* (WSB/EiJ; *domesticus* ^WSB^) and *M. m. musculus* (PWD/PhJ; *musculus* ^PWD^) were performed to generate F_1_ hybrids. A total of 305 F_2_ males were generated by mating F_1_ siblings (294 from *domesticus* ^WSB^ female×*musculus* ^PWD^ male crosses and 11 from *musculus* ^PWD^ female×*domesticus* ^WSB^ male crosses). Male F_2_s were euthanized at 70 (±5) days of age. Five sterility phenotypes were quantified: testis weight, sperm count, sperm head shape, proportion of abnormal sperm, and seminiferous tubule area (see White et al 2011 for detailed methods). The left testis was flash-frozen in liquid nitrogen upon dissection and stored at −80°. Testes from *musculus* ^PWD^ (n = 8), *domesticus* ^WSB^ (n = 8), *musculus* ^PWD^×*domesticus* ^WSB^ F_1_s (n = 6), and *domesticus* ^WSB^×*musculus* ^PWD^ F_1_s (n = 4), were dissected using the same procedure to provide controls for expression analyses. Frozen testis samples were transferred to RNAlater-ICE buffer (Invitrogen, Grand Island, NY, USA), shipped to the Max Planck Institute in Plön and stored at −80° until processing.

### Gene expression analysis

#### RNA sample preparation and microarray hybridization

We used Whole Mouse Genome Microarrays (Agilent, Waldbronn, Germany) to measure genome-wide expression in testis. This array contains 43,379 probes surveying 22,210 transcripts from 21,326 genes. We extracted RNA from testis using RNeasy kits (Qiagen, Hilden, Germany). RNA quality (RIN>8) was verified using RNA 6000 Nano kits (Agilent) on a 2100 Bioanalyzer (Agilent). We used single-color Quick-Amp Labeling Kits (Agilent) to amplify and label RNA. The yield (>2 µg) and specific activity (>9.0 pmol Cy3/µg cRNA) of labeling reactions was verified using a NanoDrop ND-1000 UV-VIS Spectrophotometer (NanoDrop, Wilmington, DE, USA). Arrays were scanned using a High Resolution Microarray Scanner (Agilent) and raw images processed using Feature Extraction Software (Agilent). Raw images and the distribution of non-uniformity outliers were visually inspected for large spatial artifacts (*e.g.* caused by buffer leakage or dust particles). We used quality control metrics from Feature Extraction protocol GE1_QCMT_Dec08.

#### Filtering and preprocessing

We mapped the 41,174 non-control probe sequences from the Whole Mouse Genome Microarray to the mouse reference genome (NCBI37, downloaded March 2011) using BLAT ([90]; minScore = 55, default settings for all other options). We excluded probes with multiple perfect matches, more than nine matches, matches to non-coding/intergenic regions only, and matches to more than one gene. A total of 36,323 probes (covering 19,742 Entrez Genes) were retained. Gene expression data have been deposited in the NCBI Gene Expression Omnibus (GSE54089).

Preprocessing of microarray data was performed using the BioConductor package Agi4x44PreProcess [91]. We used the “half” setting, which sets intensities below 0.5 to 0.5 following background subtraction, and an offset value of 50. The background signal was computed in Feature Extraction. This signal incorporates a local background measurement and a spatial detrending surface value. We used quantile normalization to normalize signal between arrays.

During preprocessing, we filtered probes based on flags from Feature Extraction. Probes with signal above background in at least 10% of samples were retained (settings: wellaboveBG = TRUE, isfound = TRUE, wellaboveNEG = TRUE). We used default settings for quality-control flags. Following preprocessing, we removed probes for which the 98^th^ percentile was <2-fold greater than background. The final data set includes 24,675 probes.

#### Differential expression, misexpression, and dominance

We performed pairwise comparisons of expression levels between *musculus* ^PWD^, *domesticus* ^WSB^ and reciprocal F_1_s using t-tests to identify differentially expressed transcripts. To account for multiple comparisons, we used a significance threshold based on the 5% false discovery rate [92]. For F_1_s, transcripts were classified as misexpressed if expression was lower or higher than both *musculus* ^PWD^ and *domesticus* ^WSB^ and there was a significant difference >0.5 in mean expression level (log_2_) between the F_1_ and both parental lines. For F_2_s, a transcript was classified as misexpressed in an individual if the expression level was greater/lower than both parental means and the difference was >two standard errors and >0.5 (log_2_) It is important to note that, using these criteria, some transcripts classified as misexpressed do not have aberrant expression patterns due to hybrid defects (*e.g.* values outside the parental range can result from transgressive segregation) and some transcripts with aberrant expression may fall within the parental range plus arbitrary fold cutoff.

To classify dominance of eQTL, we compared mean expression levels between genotypic classes at the peak marker/pseudomarker position. If the mean for heterozygotes was intermediate and >two standard errors from both homozygote means, the eQTL was classified as additive. If the heterozygous mean was <two standard errors from one homozygote mean and >two standard errors from the other, the eQTL was classified as dominant. If the heterozygous mean was outside the homozygote range and >two standard errors from the extreme homozygote mean, the eQTL was classified as over- or underdominant.

#### Ascertainment bias

Microarray probes were designed using sequences from a variety of libraries including UCSC mRNA (known genes), RefSeq, and RIKEN cDNA. All sources are based on samples from classical laboratory mouse strains, which have genomes predominantly *M. m. domesticus* in origin [93]–[95]. *M. m. domesticus* ^WSB^ is more closely related to the classical strains than *M. m. musculus* ^PWD^, raising the possibility that ascertainment bias will affect expression results. 17,508 probes were differentially expressed between *M. m. domesticus* ^WSB^ and *M. m. musculus* ^PWD^. More of these probes had higher expression in *M. m. musculus* ^PWD^ (9,265) than in *M. m. domesticus* ^WSB^ (8,243). This pattern is the opposite of what would be expected if ascertainment bias had a substantial effect. To address this issue more directly, we identified 103 probes with SNPs between the strains using the Perlegen phase 4-release of the mouse resequencing project [96]. The number of probes in this subset with higher expression in *M. m. musculus* ^PWD^ (41) was also greater than those with higher expression in *M. m. domesticus* ^WSB^ [35]. This result provides further evidence that ascertainment bias does not substantially affect measures of expression determined from this array in individuals from crosses between these strains.

### Quantitative trait locus mapping

#### Genotyping and genetic map construction

Genotyping and quality control procedures are described in White *et al* [1]. Briefly, 331 single nucleotide polymorphism (SNP) markers were genotyped using the Sequenom iPLEX MassARRAY system [97]. A total of 198 SNPs was retained for genetic mapping, following a stringent series of quality-control steps that considered parental and F_1_ controls, segregation ratios, and proportion of missing data [98]. The genetic map was estimated from the genotypes of 553 F_2_ males and females using a Carter-Falconer mapping function [99], as implemented by the *est.map* function of R/qtl [100], [101], assuming a genotyping error rate of zero [98].

#### Interval mapping

For comparison of sterility phenotype QTL and eQTL, we repeated mapping of fertility traits from White *et al* [1], excluding five F_2_ individuals (of 310) without gene expression data. Standard interval mapping was performed using the *scanone* function of R/qtl [100], [101], with identical parameter settings and data transformations as described in White *et al* [1]. Specifically, standard interval mapping (EM algorithm) was performed for all traits except abnormal sperm phenotypes, which were mapped using the extended Haley-Knott method [102].We determined genome-wide significance thresholds for autosomes from 1000 permutations; permutations for the X (15,855) were performed separately [101], [103], [104].

To identify loci causing large-scale disruptions in gene expression, we performed QTL mapping using quantitative measures of misexpression as phenotypes. Misexpression of X- and autosomal transcripts were mapped separately, on the basis of misexpression patterns in sterile F_1_ hybrids from this cross and previous studies [10]. Counts of overexpressed and underexpressed transcripts on the X and autosomes were square root transformed to improve fit to the genetic model, which assumes normality. We determined genome-wide significance thresholds for each phenotype as described above.

We mapped eQTL for 24,675 expression traits using *scanone* and the EM method. Genotype probabilities were estimated at 2 cM intervals, assuming a genotyping error rate of 0.001. We used the normal quantile ranks of gene expression values as phenotypes. The normal quantile rank transformation left all expression traits with a shared distribution, allowing us to determine genome-wide significance thresholds by permutation of a single transcript. We performed 10,000 permutations for autosomes and 158,550 for the X. In addition, we performed 360 permutations of the entire expression dataset to identify a more conservative significance threshold based on the probability of observing a single autosomal or X eQTL by chance when mapping all traits. We permuted animal IDs associated with expression data to preserve the correlation structure among transcripts.

To estimate the experiment-wide false positive rate, we translated the maximum LOD scores for each chromosome for each transcript into *P* values and determined the FDR and *q* values using the BioConductor package qvalue with a tuning parameter (λ) estimated by the “smoother” method [92].

To assess which aspects of the eQTL patterns we observed were related to hybrid sterility vs. subspecies differences unrelated to sterility, we repeated mapping eQTL mapping in 229 F_2_s without strong evidence for sterility. We identified ‘fertile’ individuals by performing a principal component analysis of four sterility phenotypes: relative right testis weight, sperm density, total abnormal sperm and seminiferous tubule area. Data for all four phenotypes were available for 286 F_2_s. Principal component one explains 99.9% variance. We included individuals >20 percentile for PC1 in the ‘fertile’ mapping analysis. Significance thresholds were 3.61 for autosomes, based on 10,000 single-transcript permutations, and 2.80 for the X chromosome, based on and 158,332 permutations.

#### Conditional mapping

Genetic interactions (epistasis) are difficult to map using standard approaches because the sample size must accommodate both the number of individuals required to recapitulate affected two-locus genotypes as well as overcome uncertainty introduced by a substantial increase in the number of statistical tests performed. Given the large (24,675) number of traits mapped in this eQTL analysis, standard approaches for identifying interactions (*e.g.* pair-scan) are expected to be underpowered to the point of futility. However, under the Dobzhansky-Muller model, hybrid sterility phenotypes are generated by negative epistasis, and each sterility locus is expected to interact with one or more additional loci. Thus, interaction partners of a hybrid-sterility QTL can be mapped by conditioning on the genotype at the candidate locus. This hypothesis-driven approach reduces the number of tests performed to the point that meaningful results can be achieved while controlling for multiple testing. Assuming *trans* hotspots indeed reflect hybrid incompatibilities, we performed conditional mapping of eQTL using genotypes at a set of 14 candidate loci as covariates, including the marker closest to the peak of each of the nine autosomal *trans* eQTL hotspots, and five markers in X chromosome *trans* hotspots. We used additional X genotypes as covariates because the large size of the *trans* hotspots on the X suggests there may be multiple underlying causative genes.

We performed conditional mapping twice for each genotype covariate, first using an additive model including an effect of the covariate, and second using a full model including an effect of the covariate and a QTL x covariate interaction term. Comparison between these models is necessary to distinguish effects of gene-gene interactions from enhanced detection of eQTL due to reduced residual variation by accounting for the effect of the covariate. The LOD score for the interaction (LOD*_i_*) is the difference between LOD scores under the full (LOD*_f_*) and additive (LOD*_a_*) models [101]. For each covariate, we determined genome-wide significance thresholds for LOD*_f_*, LOD*_a_*, and LOD*_i_* by permutation. We used the same seed for permutations (1000 autosomes, 15,855 X) under the full and additive models, enabling calculation of LOD*_i_* for each permutation. eQTL with both LOD*_f_* and LOD*_i_* above significance thresholds have significant evidence for an interaction-eQTL. Significance thresholds for each covariate are listed in Table S3.

Threshold estimates based on permutations of a single transcript for the main eQTL mapping analysis (no covariate) and for models with additive covariates were similar. For simplicity, we used 3.7 (autosomal) and 2.9 (X) as thresholds for these analyses.

#### Identification of eQTL hotspots and co-localization with sterility QTL

We defined *trans* eQTL hotspots by permutation. To preserve the distribution of eQTL across transcripts, we randomly assigned the position of each observed *trans* eQTL to a marker/pseudomarker (2 cM intervals, equal probability for each appropriate marker) on another chromosome 10,000 times for the main eQTL analysis and 1,000 times for analyses with covariates. The maximum eQTL count in each window was recorded for each permutation. Hotspots comprise sliding windows with *trans* eQTL counts greater than the 95^th^ percentile of counts for sliding windows of the same size calculated from permutations.

We tested for non-random co-localization of *trans*-eQTL hotspots with sterility phenotype QTL by permuting the positions of the *trans* hotspots, while keeping sterility QTL positions fixed at observed locations. For each permutation, positions of 12 non-overlapping hotspots of the same sizes (cM) as the observed data were drawn from the markers and pseudomarkers (2 cM step size) used for QTL mapping. Sterility QTL regions included all positions within the 1.5-LOD interval for any QTL for a sterility phenotype identified by single or multiple QTL mapping analyses in White *et al* (2011). We performed 10,000 permutations, recording the number of hotspots overlapping sterility QTL, the total number of overlapping markers/pseudomarkers, and the combined length of overlapping (cM) regions for each permutation.

### Functional annotation and identification of candidate genes

To identify classes of genes enriched among QTT, we used the DAVID functional annotation tool [43], [44], which integrates gene annotation information from several resources. Functionally related genes are clustered based on biological process, cellular compartment, molecular function, sequence features, protein domains, and protein interactions. To account for multiple comparisons, we used a significance threshold based on the false discovery rate (Benjamini) calculated within DAVID.

We identified candidate genes in *trans* hotspots and among QTT that have roles in male reproduction and/or regulation of gene expression using reviews of male fertility [17] and meiosis [105] and gene ontology (GO) terms related to male reproduction, meiosis, or the regulation of gene expression: 0001059; 0001060; 0001109; 0001121; 0003006; 0006351; 0006352; 0006353; 0006354; 0006355; 0006360; 0006366; 0006383; 0006390; 0006396; 0006412; 0007127; 0007135; 0007140; 0007285; 0009008; 0009299; 0009300; 0009302; 0009304; 0010216; 0010468; 0010608; 0010628; 0010629; 0022414; 0023019; 0030724; 0030726; 0032775; 0032776; 0036206; 0040020; 0040029; 0042793; 0043046; 0043484; 0044030; 0045132; 0045835; 0045836; 0045892; 0045893; 0048133; 0048136; 0048140; 0048515; 0048610; 0050684; 0051037; 0051257; 0051604; 0070192; 0070613; 0070920; 0080188; 0090306; 0097393; 1901148; 1901311; 2000232; 2000235; 2000241; 2000242; 2000243. Many genes identified as candidates in publications were not annotated with related GO terms, highlighting the limitations of gene ontology. Moreover, genes causing sterility might not have functions obviously related to reproduction.

## Supporting Information

Figure S1 Dominance and effect sizes of eQTL. (A) Proportions of *cis* and *trans* eQTL showing additive (add), dominant (dom), underdominant (under) and overdominant (over) effects. (B) Boxplots indicating median (horizontal lines) and interquartile range (boxes) for effects of *cis* and *trans* eQTL on the autosomes (auto) and X chromosome, measured by difference in mean expression level (log_2_) between extreme genotypes. Whiskers indicate 1.5× interquartile range. Outliers are shown as points.
(TIF)

Figure S2 Reduced clustering of *trans* eQTL mapped in a ‘fertile’ subset of F_2_s and overlap of *trans* eQTL hotspots and sterility QTL. Graphs indicate the number of *trans* eQTL mapped to 4-cM sliding windows using the fertile subset (in dark gray) and full data set (blue dashed) for each chromosome. Chromosome numbers are in bold at the bottom left of their respective plots. Dark blue boxes indicate “*trans* hotspots,” significantly enriched for *trans* eQTL. Red boxes indicate positions of sterility QTL identified previously in these mice. Abbreviations indicate phenotype: TW: relative right testis weight, DBT: distal bent tail (sperm morphology), SD: sperm density, H/T: headless/tailless (sperm), ASH: abnormal sperm head morphology, STA: seminiferous tubule area, TAS: total abnormal sperm, PBT: proximal bent tail (sperm morphology), PC1: sperm head shape principal component 1.
(TIF)

Figure S3 Clustering of ‘interaction’ eQTL identified by conditional mapping. The number of *trans* interaction eQTL for 4 cM sliding windows is plotted across the genome for each conditional mapping analysis. The position of the marker genotype used as a covariate is indicated along the y-axis. Autosomal positions are given as chromosome number, “.”, cM position. X-linked markers are given as “X” and cM position.
(TIF)

Figure S4 Overlap of interaction eQTL hotspots. Red rectangles indicate sterility QTL, with phenotype abbreviations as in Figure S2. Dark blue rectangles indicate *trans* eQTL hotspots (original mapping). ‘Interaction hotspots’ identified by conditional mapping are shown as ovals above ideograms, color coded and labeled with positions of the marker genotype covariates.
(TIF)

Figure S5 X chromosome interactions by region. Interaction network demonstrating distinct patterns for five X genotype covariates in four regions. Nodes/edges in purple involve only the proximal X region and those in green involve the distal X region. Nodes in gray show evidence for interaction with at least one proximal and one distal X covariate. Edge weight indicates the number of interaction eQTL and node size indicates total number of interactions.
(TIF)

Table S1 Misexpression of transcripts by spermatogenic cell type.
(DOCX)

Table S2 X Chromosome regions.
(DOCX)

Table S3 Significance thresholds for expression quantitative trait locus (eQTL) mapping.
(DOCX)
