# Supplementary figures and images for: Genomic Networks of Hybrid Sterility
Source: PLoS Genet. 2014 Feb 20;10(2):e1004162. doi: 10.1371/journal.pgen.1004162 (PMC3930512; doi:10.1371/journal.pgen.1004162)

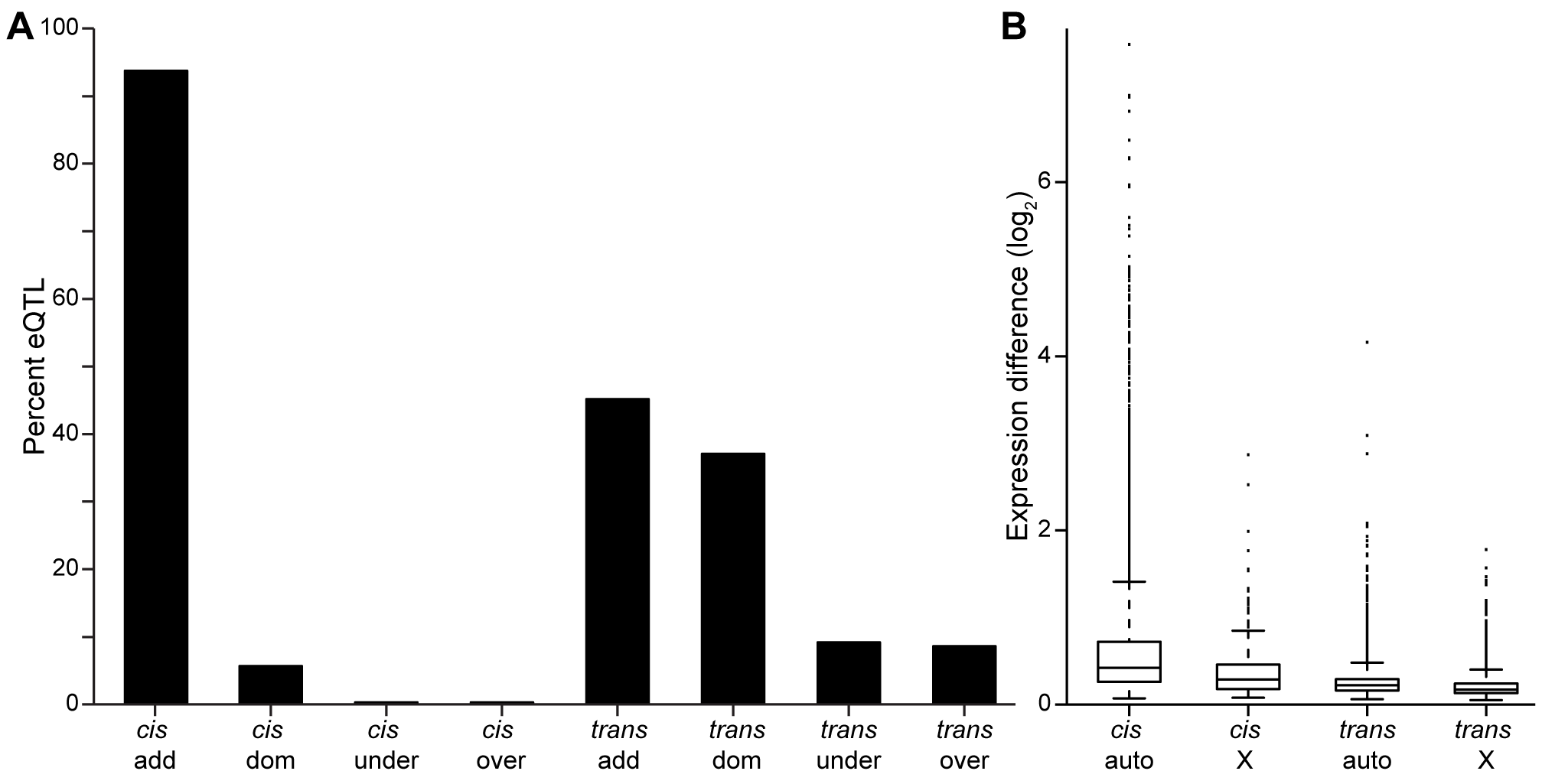

Supplement: Figure S1 — Dominance and effect sizes of eQTL. (A) Proportions of cis and trans eQTL showing additive (add), dominant (dom), underdominant (under) and overdominant (over) effects. (B) Boxplots indicating median (horizontal lines) and interquartile range (boxes) for effects of cis and trans eQTL on the autosomes (auto) and X chromosome, measured by difference in mean expression level (log2) between extreme genotypes. Whiskers indicate 1.5× interquartile range. Outliers are shown as points. (TIF) [file pgen.1004162.s001.tif]

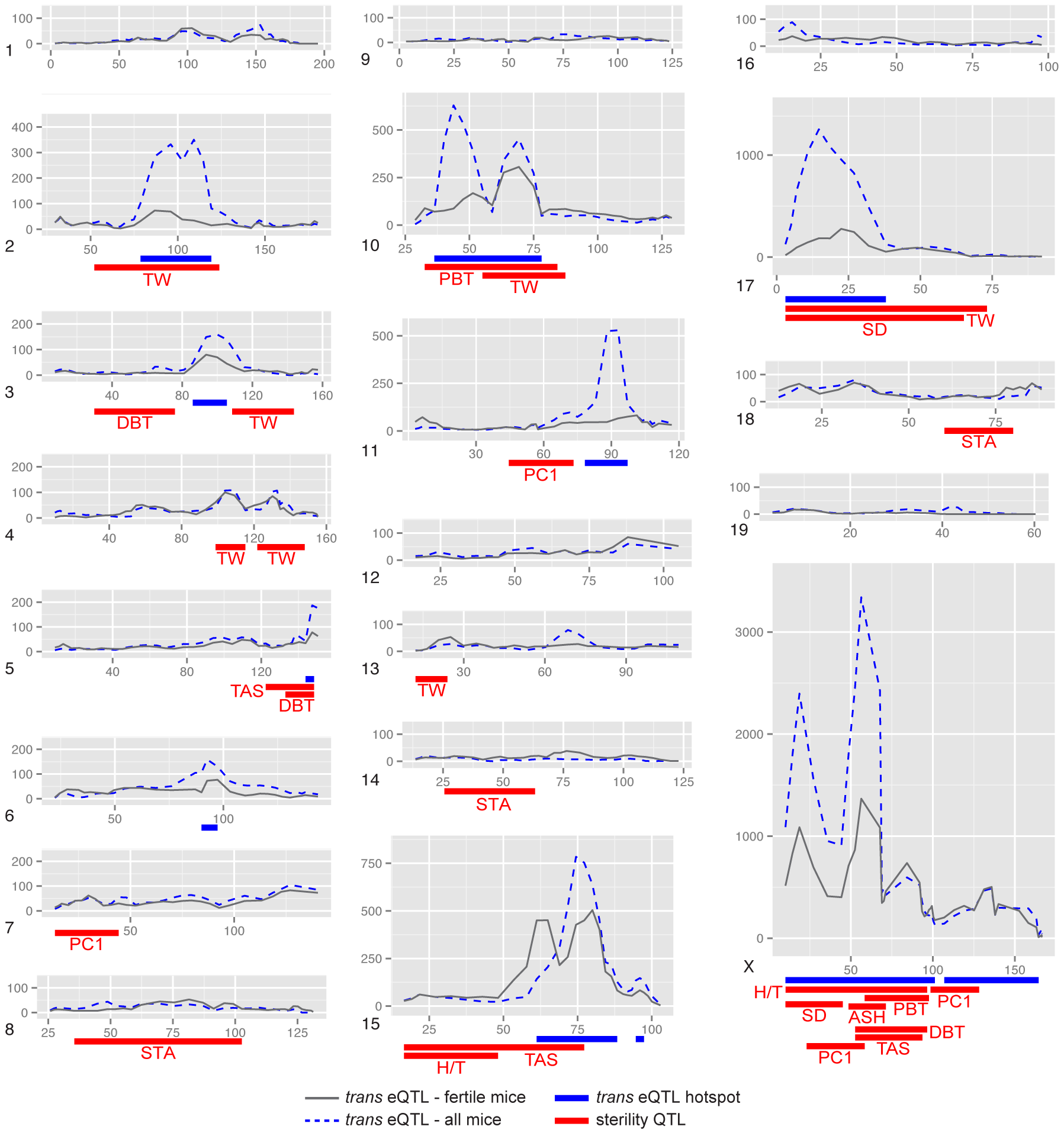

Supplement: Figure S2 — Reduced clustering of trans eQTL mapped in a ‘fertile’ subset of F2s and overlap of trans eQTL hotspots and sterility QTL. Graphs indicate the number of trans eQTL mapped to 4-cM sliding windows using the fertile subset (in dark gray) and full data set (blue dashed) for each chromosome. Chromosome numbers are in bold at the bottom left of their respective plots. Dark blue boxes indicate “trans hotspots,” significantly enriched for trans eQTL. Red boxes indicate positions of sterility QTL identified previously in these mice. Abbreviations indicate phenotype: TW: relative right testis weight, DBT: distal bent tail (sperm morphology), SD: sperm density, H/T: headless/tailless (sperm), ASH: abnormal sperm head morphology, STA: seminiferous tubule area, TAS: total abnormal sperm, PBT: proximal bent tail (sperm morphology), PC1: sperm head shape principal component 1. (TIF) [file pgen.1004162.s002.tif]

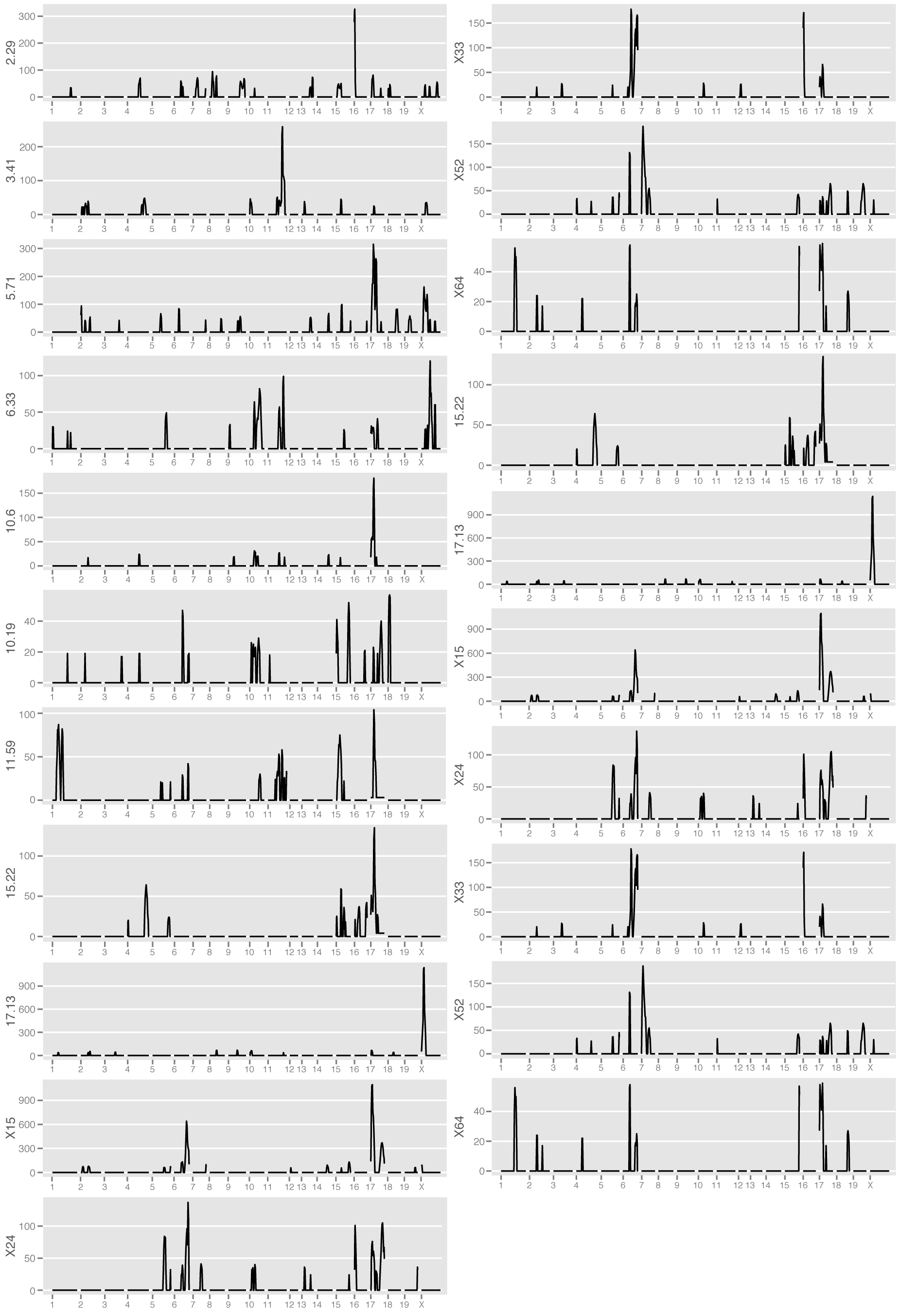

Supplement: Figure S3 — Clustering of ‘interaction’ eQTL identified by conditional mapping. The number of trans interaction eQTL for 4 cM sliding windows is plotted across the genome for each conditional mapping analysis. The position of the marker genotype used as a covariate is indicated along the y-axis. Autosomal positions are given as chromosome number, “.”, cM position. X-linked markers are given as “X” and cM position. (TIF) [file pgen.1004162.s003.tif]

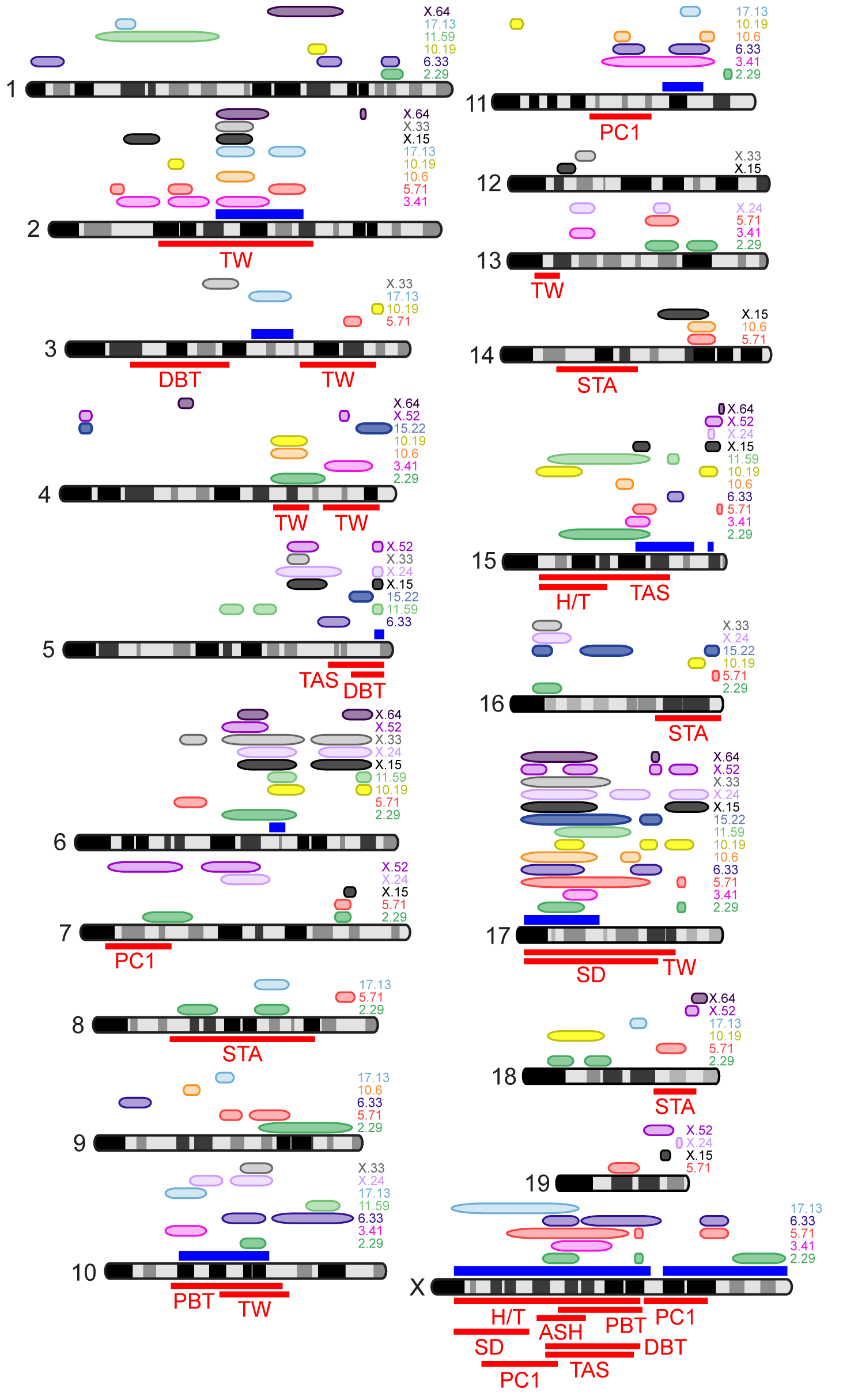

Supplement: Figure S4 — Overlap of interaction eQTL hotspots. Red rectangles indicate sterility QTL, with phenotype abbreviations as in Figure S2. Dark blue rectangles indicate trans eQTL hotspots (original mapping). ‘Interaction hotspots’ identified by conditional mapping are shown as ovals above ideograms, color coded and labeled with positions of the marker genotype covariates. (TIF) [file pgen.1004162.s004.tif]

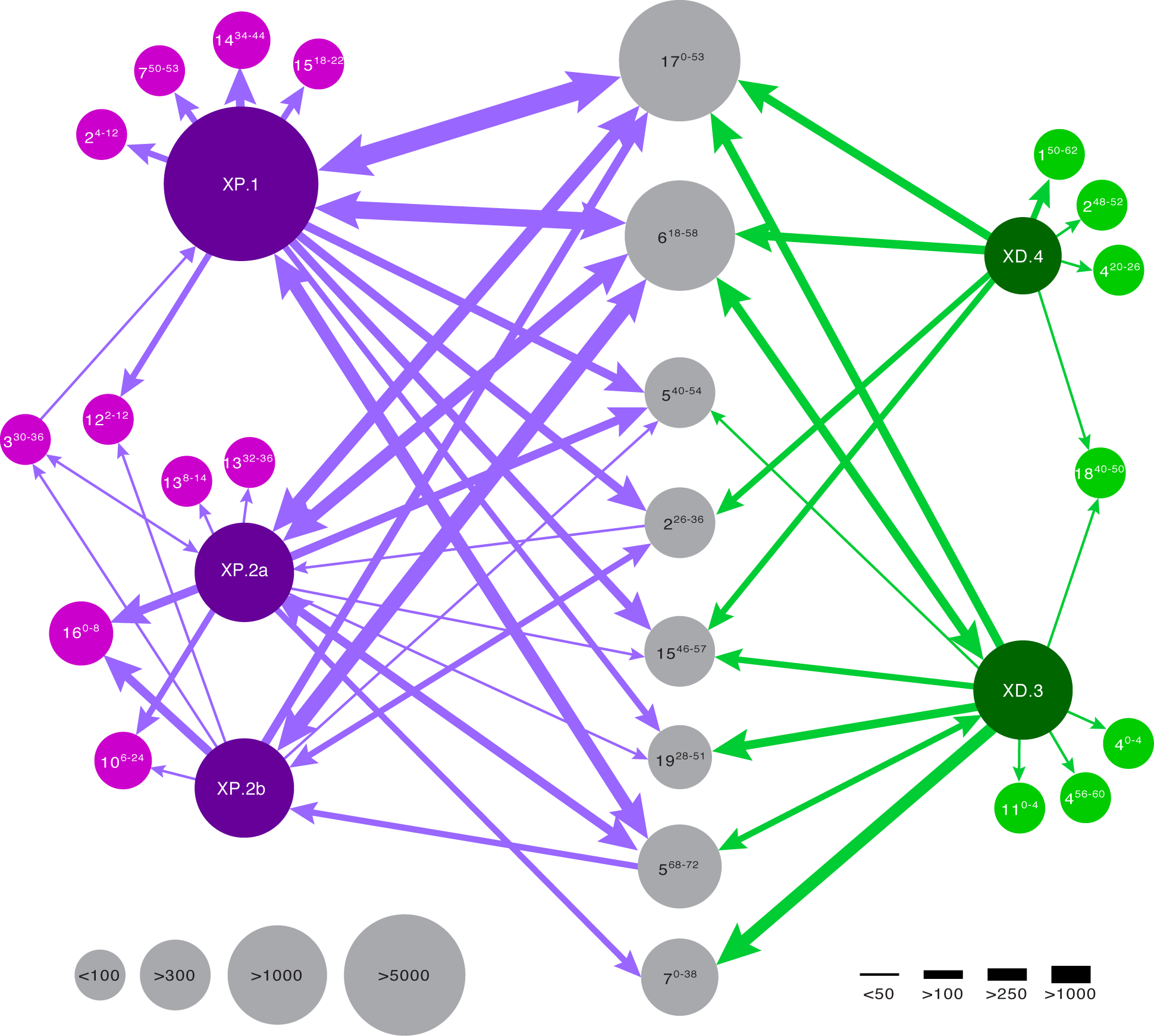

Supplement: Figure S5 — X chromosome interactions by region. Interaction network demonstrating distinct patterns for five X genotype covariates in four regions. Nodes/edges in purple involve only the proximal X region and those in green involve the distal X region. Nodes in gray show evidence for interaction with at least one proximal and one distal X covariate. Edge weight indicates the number of interaction eQTL and node size indicates total number of interactions. (TIF) [file pgen.1004162.s005.tif]
