# Supplementary material for: Genomic Networks of Hybrid Sterility
Source: PLoS Genet. 2014 Feb 20;10(2):e1004162. doi: 10.1371/journal.pgen.1004162 (PMC3930512; doi:10.1371/journal.pgen.1004162)
Supplement: Table S1 — Misexpression of transcripts by spermatogenic cell type. (DOCX) [file pgen.1004162.s006.docx]

**Table S1. Misexpression of transcripts by spermatogenic cell type.**

|  | Autosomes | | |  | X chromosome | | |
| --- | --- | --- | --- | --- | --- | --- | --- |
| Cell type^2^ | Obs | Exp | *P* |  | Obs | Exp | *P*^1^ |
| MxD F1 underexpressed |  |  |  |  |  |  |  |
| somatic | 50 | 151.3 | **1.76x10^-24^** |  | 1 | 4.2 | 0.0587 |
| mitotic | 57 | 263.8 | **2.66x10^-62^** |  | 0 | 9.6 | **7.92x10^-6^** |
| meiotic | 329 | 207.3 | **8.19x10^-19^** |  | 0 | 0 | 1 |
| post-meiotic | 805 | 139.5 | **0** |  | 16 | 3.1 | **1.91x10^-10^** |
| MxD F1 overexpressed |  |  |  |  |  |  |  |
| somatic | 214 | 97.8 | **4.00x10^-29^** |  | 40 | 22.8 | **8.01x10^-6^** |
| mitotic | 704 | 170.5 | **1.11x10^-285^** |  | 72 | 51.7 | **5.68x10^-5^** |
| meiotic | 16 | 134 | **6.72x10^-42^** |  | 0 | 0 | 1 |
| post-meiotic | 10 | 90.2 | **6.57x10^-29^** |  | 9 | 16.8 | 0.0128 |
| Underexpressed >5% F2s |  |  |  |  |  |  |  |
| somatic | 177 | 173.1 | 0.355 |  | 11 | 14.2 | 0.209 |
| mitotic | 209 | 302 | **1.90x10^-10^** |  | 20 | 32.2 | 0.00301 |
| meiotic | 280 | 237.3 | 0.00111 |  | 0 | 0 | 1 |
| post-meiotic | 451 | 159.7 | **5.58x10^-105^** |  | 22 | 10.4 | **5.54x10^-5^** |
| Overexpressed >5% F2s |  |  |  |  |  |  |  |
| somatic | 272 | 145.1 | **5.26x10^-26^** |  | 33 | 24.2 | 0.0115 |
| mitotic | 543 | 253.1 | **5.21x10^-75^** |  | 78 | 55 | **9.91x10^-06^** |
| meiotic | 74 | 198.9 | **6.83x10^-28^** |  | 0 | 0 | 1 |
| post-meiotic | 79 | 133.8 | **2.68x10^-8^** |  | 13 | 17.9 | 0.103 |

^1^hypergeometric test. Values significant at Bonferroni-corrected *P<* 0.00045 are in bold.

^2^spermatogenic cell-type classifications based on Chalmel et al. 2007. SO: somatic; MI: mitotic; ME: meiotic; PM: postmeiotic
