# Supplementary material for: Genomic Networks of Hybrid Sterility
Source: PLoS Genet. 2014 Feb 20;10(2):e1004162. doi: 10.1371/journal.pgen.1004162 (PMC3930512; doi:10.1371/journal.pgen.1004162)
Supplement: Table S2 — X Chromosome regions. (DOCX) [file pgen.1004162.s007.docx]

**Table S2. X Chromosome regions.**

|  |  | |  |  | | *dom* allele high | | | | | | | |  | | *mus* allele high | | | | | | | |  | |  | |  | |  |
| --- | --- | --- | --- | --- | --- | --- | --- | --- | --- | --- | --- | --- | --- | --- | --- | --- | --- | --- | --- | --- | --- | --- | --- | --- | --- | --- | --- | --- | --- | --- |
| Hotspot region | | **Position cM** | **Position Mb** | | ***trans* eQTL** | | **MxD F1 underexp** | | **MxD F1 overexp** | | **Pos. cor RRTW** | | **Neg. cor RRTW** | |  | | **MxD F1 underexp** | | **MxD F1 overexp** | | **Pos. cor RRTW** | | **Neg. cor RRTW** | | **Sterile Allele** | | **Marker position covariate (cM)** | | **Sterility QTL** | |
| Proximal |  | |  |  | |  | |  | |  | |  | |  | |  | |  | |  | |  | |  | |  | |  | |  |
| 1 | 0-16 | | 10.2-67.8 | 911-3,340 | | 29-63 | | 0-1 | | 83-98 | | 0-3 | |  | | 0-1 | | 16-40 | | 0-3 | | 90-96 | | M | | 14.98 | | ASH@2.5^M, A^; ASH@10.3^M, A^; ASH@19^M, A^; ASH@25.5^M, A^; ASH^MOL, C^; SC@15.1^M, A^; TW@13.3^M, A^; TW@24^M, A^ | |  |
| 2 | 18-42 | | 68.9-101.2 | 133-596 | | 0-19 | | 0-3 | | 28-66 | | 6-37 | |  | | 1-5 | | 1-16 | | 7-50 | | 21-77 | | M | | 24.51; 33.39 | | ASH^M, B^; ASH@42.7^M, A^; OFF^M, B^; SC^M, B^ ;TW^M, B^ | |  |
| Total | 0 - 42 | | 10.2-101.2 | 8,286 | | 36.0 | | 0.1 | | 83.2 | | 4.8 | |  | | 0.2 | | 28.8 | | 4.1 | | 87.9 | |  | |  | |  | |  |
| Distal |  | |  |  | |  | |  | |  | |  | |  | |  | |  | |  | |  | |  | |  | |  | |  |
| 3 | 44-56 | | 106.9-137.9 | 143-487 | | 1-7 | | 2-6 | | 16-37 | | 37-58 | |  | | 1-12 | | 1-5 | | 55-74 | | 9-25 | | D | | 52.19 | | ASH@49^M, A^; SC@67^M, A^; TW@59.5 ^M, A^ | |  |
| 4 | 58-66 | | 140-164.4 | 32-299 | | 0-3 | | 3-18 | | 0-5 | | 80-96 | |  | | 20-39 | | 0-1 | | 80-95 | | 0-5 | | D | | 63.69 | | ASH@73.3^M, A^; ASH^MOL, C^; TW^MOL,C^ | |  |
| Total | 44 - 66 | | 106.9-164.4 | 1,180 | | 2.7 | | 5.6 | | 14.9 | | 64.0 | |  | | 15.5 | | 1.4 | | 74.2 | | 10.4 | |  | |  | |  | |  |

Values listed are ranges for 4 cM sliding windows with centers indicated in “Position cM” column. Values in “*dom* allele high” and “*mus* allele high columns” indicate the low- and high-end of percentages of QTT for sliding windows fitting the criterion.

ASH: abnormal sperm head; SC: sperm count; TW: testis weight, OFF: number offspring, M: *musculus* allele sterile, MOL: *M. m. molossinus* allele sterile

^A^Good et al 2008 Genetics

^B^Storchova et al 2004 Mamm Gen

^C^Oka 2004
