# Supplementary material for: Genomic Networks of Hybrid Sterility
Source: PLoS Genet. 2014 Feb 20;10(2):e1004162. doi: 10.1371/journal.pgen.1004162 (PMC3930512; doi:10.1371/journal.pgen.1004162)
Supplement: Table S3 — Significance thresholds for expression quantitative trait locus (eQTL) mapping. (DOCX) [file pgen.1004162.s008.docx]

**Table S3. Significance thresholds for expression quantitative trait locus mapping.**

|  |  | **5% significance thresholds autosomes** | | |  | **5% significance thresholds**  **X chromosome** | | |  |  |
| --- | --- | --- | --- | --- | --- | --- | --- | --- | --- | --- |
| **Covariate (marker)** | **location** | **LOD*_a_*** | **LOD*_f_*** | **LOD*_i_*** |  | **LOD*_a_*** | **LOD*_f_*** | **LOD*_i_*** |  | **Minimum eQTL count/4 cM window**  ***trans* hotspots** |
| none |  | 3.6 | - | - |  | 2.8 |  |  |  | 155 |
| NES09108608 | Chr 2@29cM | 3.6 | 4.4 | 1.9 |  | 2.8 | 3.3 | 2.3 |  | 30 |
| NES13927401 | Chr 3@41cM | 3.6 | 4.7 | 2.1 |  | 2.8 | 3.5 | 2.5 |  | 21 |
| NES10364112 | Chr 5@71cM | 3.6 | 4.5 | 1.9 |  | 2.9 | 3.4 | 2.3 |  | 36 |
| NES11922718 | Chr 6@33cM | 3.6 | 4.8 | 2.3 |  | 2.7 | 3.5 | 2.6 |  | 20 |
| NES16893219 | Chr 10@6cM | 3.5 | 4.7 | 2.2 |  | 3.0 | 3.6 | 2.6 |  | 16 |
| NES14174531 | Chr 10@19cM | 3.7 | 4.8 | 2.2 |  | 2.7 | 3.5 | 2.6 |  | 16 |
| NES08577121 | Chr 11@59cM | 3.7 | 4.7 | 2.1 |  | 2.8 | 3.5 | 2.5 |  | 19 |
| NES17019164 | Chr 15@22cM | 3.6 | 4.9 | 2.3 |  | 2.9 | 3.7 | 2.8 |  | 17 |
| NES16574315 | Chr 17@13cM | 3.6 | 4.5 | 1.8 |  | 2.8 | 3.4 | 2.3 |  | 35 |
| NES12384176 | Chr X@15cM | 3.6 | 4.7 | 2.0 |  | 2.8 | 3.3 | 2.2 |  | 55 |
| NES09680385 | Chr X@24cM | 3.6 | 4.7 | 2.2 |  | 2.8 | 3.5 | 2.4 |  | 23 |
| NES09660234 | Chr X@33cM | 3.5 | 5.0 | 2.5 |  | 2.9 | 3.6 | 2.6 |  | 19 |
| NES09767342 | Chr X@52cM | 3.5 | 4.6 | 2.1 |  | 2.8 | 3.5 | 2.4 |  | 26 |
| NES11023996 | Chr X@64cM | 3.6 | 4.7 | 2.2 |  |  | 2.8 | 2.4 |  | 16 |
